# Adverse drug events in cost-effectiveness models of pharmacological interventions for diabetes, diabetic retinopathy, and diabetic macular edema: a scoping review

**DOI:** 10.11124/JBIES-23-00511

**Published:** 2024-07-29

**Authors:** Mari Pesonen, Virpi Jylhä, Eila Kankaanpää

**Affiliations:** 1 Department of Health and Social Management, University of Eastern Finland, Kuopio, Finland; 2 Finnish Centre for Evidence-Based Health Care: A JBI Centre of Excellence, Helsinki, Finland; 3 Research Centre for Nursing Science and Social and Health Management, Kuopio University Hospital, Wellbeing Services County of North Savo, Finland

**Keywords:** adverse drug events, cost-effectiveness analysis, diabetes, diabetic macular edema, diabetic retinopathy

## Abstract

**Objective:**

The objective of this review was to examine the role of adverse drug events (ADEs) caused by pharmacological interventions in cost-effectiveness models for diabetes mellitus, diabetic retinopathy, and diabetic macular edema.

**Introduction:**

Guidelines for economic evaluation recognize the importance of including ADEs in the analysis, but in practice, consideration of ADEs in cost-effectiveness models seem to be vague. Inadequate inclusion of these harmful outcomes affects the reliability of the results, and the information provided by economic evaluation could be misleading. Reviewing whether and how ADEs are incorporated in cost-effectiveness models is necessary to understand the current practices of economic evaluation.

**Inclusion criteria:**

Studies included were published between 2011–2022 in English, representing cost-effectiveness analyses using modeling framework for pharmacological interventions in the treatment of diabetes mellitus, diabetic retinopathy, or diabetic macular edema. Other types of analyses and other types of conditions were excluded.

**Methods:**

The databases searched included MEDLINE (PubMed), CINAHL (EBSCOhost), Scopus, Web of Science Core Collection, and NHS Economic Evaluation Database. Gray literature was searched via the National Institute for Health and Care Excellence, European Network for Health Technology Assessment, the National Institute for Health and Care Research, and the International Network of Agencies for Health Technology Assessment. The search was conducted on January 1, 2023. Titles and abstracts were screened for inclusion by 2 independent reviewers. Full-text review was conducted by 3 independent reviewers. A data extraction form was used to extract and analyze the data. Results were presented in tabular format with a narrative summary, and discussed in the context of existing literature and guidelines.

**Results:**

A total of 242 reports were extracted and analyzed in this scoping review. For the included analyses, type 2 diabetes was the most common disease (86%) followed by type 1 diabetes (10%), diabetic macular edema (9%), and diabetic retinopathy (0.4%). The majority of the included analyses used a health care payer perspective (88%) and had a time horizon of 30 years or more (75%). The most common model type was a simulation model (57%), followed by a Markov simulation model (18%). Of the included cost-effectiveness analyses, 26% included ADEs in the modeling, and 13% of the analyses excluded them. Most of the analyses (61%) partly considered ADEs; that is, only 1 or 2 ADEs were included. No difference in overall inclusion of ADEs between the different conditions existed, but the models for diabetic retinopathy and diabetic macular edema more often omitted the ADE-related impact on quality of life compared with the models for diabetes mellitus. Most analyses included ADEs in the models as probabilities (55%) or as a submodel (40%), and the most common source for ADE incidences were clinical trials (65%).

**Conclusions:**

The inclusion of ADEs in cost-effectiveness models is suboptimal. The ADE-related costs were better captured than the ADE-related impact on quality of life, which was most pronounced in the models for diabetic retinopathy and diabetic macular edema. Future research should investigate the potential impact of ADEs on the results, and identify the criteria and policies for practical inclusion of ADEs in economic evaluation.

**Supplemental digital content:**

A Finnish-language version of the abstract of this review is available: http://links.lww.com/SRX/A68.

## Introduction

The role of health technology assessment is an integral part of determining publicly funded treatment selection in health care. For example, pharmacological treatments entering the health care market need to demonstrate their value for money, namely, that their achieved benefit is worth the accrued costs.

Health technology assessment entails a comprehensive review of clinical evidence and an economic evaluation. Because pharmaceutical treatments have potential for both benefit and harm, a prerequisite for marketing authorization is to show a positive benefit-harm ratio. This, however, is insufficient for health care funding organizations’ recommendations in publicly funded treatment selection; economic evaluation needs to demonstrate how these benefits and harms are translated into value in both clinical and economic terms. A cost-effectiveness analysis, including cost-utility analysis that measures effectiveness in terms of quality-adjusted life years (QALYs), is a common way of performing economic evaluation in health care, enabling a comparison of the costs and the effectiveness between comparators. Use of a modeling framework provides the means for extrapolating the outcomes for a longer follow-up period, thus giving a better view on the long-term consequences of an intervention. Commonly used model types include decision trees, cohort, and individual state-transition models (eg, Markov model, microsimulation), and discrete event simulation models, all of which have their own advantages and disadvantages.

Decision trees are simple to build and modify, and they are especially useful in short time horizons where estimation of outcomes is straightforward. State-transition models allow modeling for longer time frames when probabilities vary over time. The decision problem is conceptualized as a series of discrete health states, and the transitions between the health states define progression over time. Individual modeling is preferred over cohort modeling, if the decision problem requires a large number of health states. Discrete event simulation models, on the other hand, are applicable when the decision problem involves interactions among individuals. These types of models consider time as continuous rather than discrete periods. The decision problem determines the appropriate model type.^1^ For pharmaceutical therapies, state-transition models are the most applied modeling methods.

Adverse events are harmful, negative outcomes associated with any medical care, whereas adverse drug events (ADEs) are similar negative outcomes but associated only with drug therapies.^2,3^ These events exist in 2 types: those caused by an error (preventable) and those that occur despite proper treatment (nonpreventable). Adverse events or ADEs do not necessarily have a causal relationship with the treatment, rather, the cause may be preventable and related to anything regarding the medication process (eg, drug administration, drug distribution). Other forms of ADEs are adverse drug reactions and adverse effects, which are always causally related to the drug itself and, therefore, inherent in the drug.^4^

When performing cost-effectiveness analysis for pharmacological interventions, all the relevant health effects and costs related to the interventions and the condition are important to consider. The principal result for cost-effectiveness is the ratio of the difference in costs to the difference in acquired effectiveness (incremental cost-effectiveness ratio). ADEs affect both effectiveness and costs of interventions. From the perspective of health care, the effectiveness of an intervention comprises the expected number of life years adjusted for changes in quality of life, commonly measured in terms of QALYs. Quality of life includes both the improvements due to alleviation or prevention of morbidity and the impact of ADEs. The total costs are calculated by adding direct health care costs plus costs related to treatment of adverse effects, and then subtracting the cost savings due to intervention.^5^ The societal perspective is wider and includes indirect costs related to the intervention (eg, informal care, social services). Societal perspective is usually the recommended perspective for economic evaluation,^6^ although some variability in the recommendations also exists.^7^

Guidelines for economic evaluation recognize the significance of ADEs, both in the costs and effectiveness estimates in the analysis,^8–11^ but despite their importance, consideration of ADEs in cost-effectiveness models seems to be vague. This was observed in a review of health technology assessment reports commissioned by the National Institute for Health and Care Research and published between 2004 and 2007 for different medical conditions and mainly for pharmaceuticals.^12^ According to this survey, only 54% of the decision models in health technology assessments included adverse effects, and of the models that included them, only 60% considered them in both clinical and cost parameters. No clear relationship existed between inclusion of adverse effects in the model and, for example, therapeutic area, type of intervention, or the model type.

The role of adverse events has been examined in some specific therapy areas. Lu *et al.*,^13^ for example, investigated the use of disutilities of adverse events (the quality of life parameters) in cost-utility analyses of cancer drug therapies. The authors concluded that 54% of the models included disutilities of adverse events, and only 15% of the analyses provided a justification for inclusion and exclusion of disutilities of adverse events. Heather *et al.* ^14^ concluded in their systematic review for decision analytic models of anti–tumor necrosis factor drugs that the models were unable to systematically consider the direct costs and consequences of ADEs. Likewise, Pearce *et al.* ^15^ reviewed economic evaluations for antineoplastic drugs in patients with solid tumor cancers and discovered that current models may underestimate the effect of ADEs in analyses. Seemingly, there are some disparities in the current application of adverse events and ADEs to the decision analytic models compared to recommendations for economic evaluation. Although this observation appears to be common for different therapy areas, some variability is possible and models for some therapy areas may capture ADEs better than others.

Diabetes mellitus (DM) is a condition traditionally defined by chronically elevated blood sugar levels. This hyperglycemia is associated with organ damage and dysfunction in the retina, kidney, blood vessels, heart, and nerves. Different types of DM exist, of which type 1 and type 2 are the most common. The prevalence of DM worldwide in 2021 was 6.1%, and continues to increase.^16,17^ The pharmacotherapy of DM includes hypoglycemics, such as insulins, sodium-glucose cotransporter-2 inhibitors, glucagon-like peptide-1 receptor agonists, metformin, dipeptidyl peptidase-4 inhibitors, thiazolidinediones, and sulfonylureas. Depending on the medication, their ADEs include nausea, genital infections, urinary tract infections, diarrhea, hypoglycemia, headaches, and weight gain.^18^

The first complications of DM include diabetic retinopathy (DR), which is a disease of the retina causing significant vision loss and visual acuity weakening. Diabetic macular edema (DME) is the most common cause of DR-related vision loss causing abnormal thickening in the retina yielding to visual acuity weakening or loss.^19,20^ The estimated prevalence within the DM population is 34.6% for DR and 6.8% for DME.^21^ Like DM, DR is treated with hypoglycemics; however, the treatment of DME includes intravitreal anti–vascular endothelial growth factor and/or corticosteroid injections, which can cause ADEs such as cataracts, intraocular pressure rise, retinal detachment, endophthalmitis, and vitreous hemorrhage. These ocular ADEs differ in terms of severity and prevalence, for example, endophthalmitis is severe but rare, whereas vitreous hemorrhage is more common but less severe.^22,23^

In a review of economic models for age-related macular degeneration, adverse (drug) events were inconsistently included in the models,^24^ which most likely applies to models for DR and DME as well. Economic models for DM may also have inconsistent practices for inclusion of ADEs,^25,26^ but because of the broad literature and rigorously validated and advanced models (eg, see Pesonen *et al.* ^27^ in Appendix I), they potentially could better capture ADEs compared with models of ocular conditions. The incorporation of ADEs in the cost-effectiveness analyses across different diseases is an important topic that lacks systematic research.

A preliminary search of PROSPERO, MEDLINE, the Cochrane Database of Systematic Reviews, and *JBI Evidence Synthesis* was conducted, and at the time our protocol was developed,^28^ no other current or in-progress scoping reviews or systematic reviews on the topic were identified.

To capture the potential differences in incorporation of ADEs in different therapy areas, the purpose of this review was to systematically investigate a large sample of cost-effectiveness models and their incorporation of ADEs, thereby informing the practices of economic evaluation. The aim was to examine whether ADEs have been included in the model-based cost-effectiveness analyses of pharmacological interventions in patients with DM, DR, and DME. By pharmacological intervention, we refer to a treatment with drug therapies. Because of the scarce literature on the topic, a scoping review was deemed a feasible methodology for mapping the relevant evidence and informing future research. The objective of this scoping review was to explore the role of ADEs caused by pharmacological interventions in cost-effectiveness analyses for DM, DR, and DME.

## Review questions

- Are ADEs included in the model-based cost-effectiveness analyses conducted for pharmacological interventions in DM, DR, and DME?
- If so, how are these ADEs incorporated in the analyses?

## Inclusion criteria

### Participants

This review considered studies that included patients receiving pharmacological intervention for type 1 (T1DM) and/or type 2 diabetes (T2DM), DR, or DME. All other conditions were excluded. The included pharmacological therapies were indicated for these conditions. If the pharmacological therapy was indicated for conditions other than the included conditions (eg, pharmacological therapies only for cardiovascular diseases), the study was excluded. Studies where the pharmacological treatments received by the included patients were not indicated for the included conditions were excluded. No specific age range for the participants was applied.

### Concept

This review examined the inclusion of ADEs caused by pharmacological interventions in cost-effectiveness analyses. Therefore, ADEs are the outcomes of the studies but also a component of this scoping review’s concept. The concept did not limit the search, as the exclusion of ADEs from the cost-effectiveness analyses was also a valid result.

The concept consisted of 2 parts: i) whether ADEs were incorporated in the cost-effectiveness analyses and ii) how ADEs were incorporated in the cost-effectiveness analyses. Specifically, the *how* part of the concept explored whether ADEs were incorporated in cost estimates and/or quality of life estimates and what the rationale was for their potential omission. Also, the practical execution of the incorporation of ADEs was examined (eg, different health states for ADEs in the model/direct inclusion in the expected costs and QALYs; sources of the costs, disutilities, and incidences of ADEs), the reasoning behind the inclusion of ADEs (eg, thresholds for the severity or incidence of ADEs), and the possible discussion on ADEs and their impact on the results of the analysis.

### Context

The context did not include any restrictions regarding geography, culture, or race.

### Types of sources

This scoping review considered cost-utility analyses that measure effectiveness in terms of QALYs (often also termed cost-effectiveness analyses), using an economic modeling framework. These types of analyses were chosen because they evaluate a treatment’s impact on health-related quality of life. Only cost-effectiveness analyses that evaluated at least 1 pharmacological intervention were included; therefore, cost-effectiveness analyses that did not include pharmacological interventions were excluded. To make the inclusion criteria more precise, cost-effectiveness analyses comparing dosing regimens rather than distinct interventions were excluded from the review, which is a deviation from the protocol. No limitations for the model time horizon were applied, thus the review included both short-term models (defined by the authors as 0 to 10 years) and long-term models (defined by the authors as > 10 years).

The cost-effectiveness analyses had to report the incremental cost-effectiveness ratio to be included in this review, which is the most commonly used outcome for economic evaluations for pharmaceuticals. Moreover, in accordance with guidelines for health technology assessments, the included analyses needed to report QALYs to be included in the review, so technically the analyses included in this review are cost-utility analyses. In health economic literature, cost-utility analyses are usually termed as cost-effectiveness analysis; therefore this review uses the term *cost-effectiveness analysis* but also considers the term *cost-utility analysis* in the inclusion criteria.

## Methods

This scoping review was conducted in accordance with the JBI methodology for scoping reviews^28^ and was reported in line with the Preferred Reporting Items for Systematic Reviews and Meta-Analyses extension for Scoping Reviews (PRISMA-ScR).^29^ This review follows an a priori protocol.^27^

### Search strategy

The search strategy aimed to locate published, peer-reviewed cost-effectiveness analyses. Sources of gray literature were also searched.

An initial limited search of MEDLINE (PubMed) was undertaken to identify articles on the topic in February 2022. The text words contained in the titles and abstracts of relevant articles, and the MeSH terms related to the keywords, were used to develop a full search strategy. The search strategy, including all identified keywords and index terms, was adapted for each included information source. A second search was undertaken across these sources on January 1, 2023. The full search strategies from the databases are provided in Appendix I.

Only studies published in English were included because most of the cost-effectiveness and cost-utility analyses are published in English. Studies published from January 1, 2011, to December 31, 2022, were included, as relatively new data are needed in order to develop an overview of the current practices in economic evaluation. The databases that were searched included MEDLINE (Ovid), CINAHL (EBSCOhost), Scopus, Web of Science Core Collection, and NHS Economic Evaluation Database. Sources of gray literature included the National Institute for Health and Care Excellence (NICE), European Network for Health Technology Assessment (EUnetHTA), the National Institute for Health and Care Research, and the International Network of Agencies for Health Technology Assessment (INAHTA) technology appraisals. Systematic reviews including cost-effectiveness models that met the inclusion criteria of this scoping review were identified from the search and were screened for additional cost-effectiveness analyses.

### Study selection

**Figure 1:**
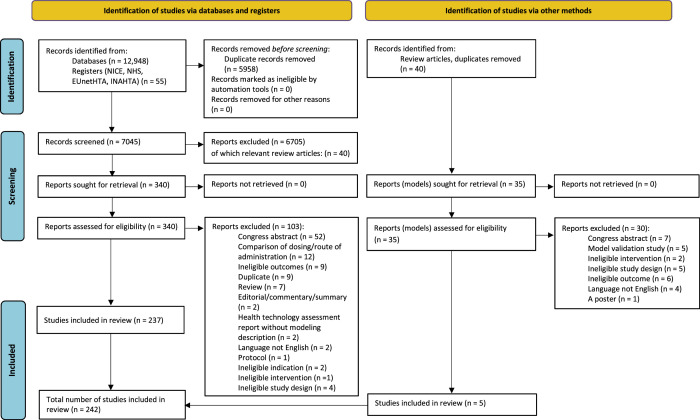
Search results and study selection and inclusion process^30^

Following the search, all identified records were collated and uploaded into Covidence (Veritas Health Innovation, Melbourne, Australia) and duplicates removed. Titles and abstracts were screened by all 3 members of the review team, with 2 reviewers independently screening each record for assessment against the inclusion criteria. Potentially relevant papers were retrieved in full. Full-text studies were screened by all 3 reviewers, with 2 reviewers per report. Full-text studies that did not meet the inclusion criteria were excluded, and reasons for their exclusion are provided in Figure 1 and Appendix II. Any disagreements that arose between the reviewers were resolved through discussion or with the third reviewer.

### Data extraction

Data were extracted from papers included in the scoping review independently by 2 reviewers using a data extraction tool developed by the reviewers. The information collected included the treatment comparison, type of modeling used, time horizon, perspective of the analysis, applied discount rate, source of efficacy results, patient-reported outcome measure (PROM) used, and the result (incremental cost-effectiveness ratio and narrative conclusion) of the analysis.

The data extraction tool in the a priori protocol^27^ was slightly modified for this scoping review (Appendix III). The data extracted included specific details about the population, concept, and context relevant to the review question. The modifications for the data extraction tool for population included specifying population according to the condition (ie, T1DM, T2DM, DR, or DME); removing *type of analysis* because all the analyses in this scoping review are cost-utility analyses per se; removing *costs included* because the relevant costs for ADEs were collected elsewhere in the instrument, and adding *source of efficacy results* because it brings valuable information on whether the source of safety results (ADEs) is different from the source of the efficacy results.

In addition, the data extraction flow for *concept* was altered so that the first decision node was whether the analysis included ADEs (“yes”), partly included ADEs (“partly”), or did not include ADEs (“no”). This grouping of analyses was based on a choice of the reviewers. We considered an analysis to include ADEs (“yes”) if more than 2 of the ADEs mentioned in the efficacy studies and/or summary of product characteristics were considered in the analysis. We considered “partly” to mean that 1 or 2 of the possible ADEs were included and other possible ADEs omitted. “No” meant that no ADEs were included in the analysis. If the decision was “yes,” the data extraction instrument collected the ADEs that were included, the source of the incidence of ADEs, how ADEs were included in the model, whether ADEs were considered in cost estimates (yes/partly/no), the source of ADE-related costs, whether ADEs were considered in quality-of-life estimates (yes/partly/no), the source and PROM used for ADE-related quality-of-life estimates, and additional details on incorporation of ADEs. In the context of costs and quality-of-life estimates, “partly” meant that the included ADEs considered ADE-related costs and impact on quality of life in varying manners in the model. If the decision was “partly,” the data extraction instrument also considered a justification for why inclusion was deemed as partly and not as a full inclusion. If the decision was “no,” data were extracted regarding whether ADEs were reported in the underlying efficacy study for the cost-effectiveness analysis or cost-utility analysis (yes/no) and the reasoning for not including ADEs in the analysis (free text).

After 2 reviewers piloted the data extraction form with 5 included studies, 1 reviewer carried out data extraction for eligible studies and another reviewer independently extracted data for 20% of the included studies using the same data extraction template. These 20% of the studies were randomly selected using a random number generator.

### Data analysis and presentation

Characteristics of included studies are presented in tabular format, including the information described in the data extraction form. The characteristics are further presented in relation to the first review question (whether ADEs are incorporated). The first review question will be further elaborated on per condition in tabular format. Within the tables and the narrative summary, we examined whether the attributes of sources (eg, disease, time horizon, PROM, publication year) could affect the inclusion or exclusion of ADEs in cost-effectiveness and cost-utility analyses. Results are also presented in a tabular format in relation to the second review question (how ADEs are incorporated). A narrative summary accompanies all the tabulated results. The full data extraction is available as supplemental digital content: http://links.lww.com/SRX/A52.

## Results

### Study inclusion

Records identified from databases and registers included 13,003 papers. After 5958 duplicate records were removed, a total of 7045 records were screened. During screening, 6705 records were excluded based on title and abstract. Of these, 40 review articles were stored for further hand-searching for potential eligible studies. A total of 340 reports were retrieved for full-text review, of which 103 were excluded. Reasons for exclusion of full-text reports are presented in Figure 1 and Appendix II. A total of 237 studies from the database and register search were included in the review. Additionally, the 40 systematic reviews excluded during screening were hand-searched, with 35 unique reports identified and full texts retrieved. Of these, 30 reports were excluded (see Figure 1 and Appendix II), and 5 additional studies were included in the review. In total, 242 studies were included in this scoping review.^31–272^

### Characteristics of included studies

The cost-effectiveness analyses included studies published between January 1, 2011 and December 31, 2022. The sample was inclined to more recently published analyses, as analyses published in years 2019 to 2022 accounted for 48% of included analyses. The majority of analyses (83%) originated from the UK, US, China, and Europe. T2DM was the most common indication (86%) for the included analyses; T1DM, DME, and DR accounted for 10%, 9%, and 0.4%, respectively. Health care payer perspective was used in 88% in the included analyses and societal perspective in 13% of the analyses; some of the analyses (2%) included both perspectives. Most of the analyses (75%) had a time horizon of 30 years or more, although a time horizon of 1 year or less was included in 8% of the analyses.

The most common model type reported was a simulation model (57%), followed by a Markov model (18%), a microsimulation model (6%), and a discrete event simulation model (5%). Models without specification accounted for 8% of the included models. In total, studies reported 16 individual models, 50 models self-made by authors, 2 models from NICE, and 11 health technology assessment reports by NICE or the Canadian Agency for Drugs and Technologies in Health (CADTH).

The characteristics of included analyses are presented in Table 1 according to cost-effectiveness analyses that included ADEs, cost-effectiveness analyses that partly included ADEs (ie, 1 or 2 ADEs included, although other relevant ones exist), and cost-effectiveness analyses that excluded ADEs. Other information, such as treatment comparisons, source of efficacy results, and PROMs used, are available in Appendix IV and supplemental digital content: http://links.lww.com/SRX/A52.

**Table 1:** Characteristics of included studies that used cost-effectiveness analyses to evaluate pharmacological interventions and their inclusion of adverse drug events

Characteristics	CEAs (N=242)	CEAs that included ADEs (n=62, 26%)	CEAs that partly included ADEs (n=148, 61%)	CEAs that did not include ADEs (n=32, 13%)
*Year of publication*				
2011	8 (3%)	2 (25%)	6 (75%)	0 (0%)
2012	19 (8%)	3 (16%)	11 (58%)	5 (26%)
2013	10 (4%)	4 (40%)	6 (60%)	0 (0%)
2014	11 (5%)	3 (27%)	8 (73%)	0 (0%)
2015	17 (7%)	7 (41%)	9 (53%)	1 (6%)
2016	21 (9%)	7 (33%)	11 (52%)	3 (14%)
2017	22 (9%)	3 (14%)	18 (82%)	1 (5%)
2018	20 (8%)	4 (20%)	15 (75%)	1 (5%)
2019	28 (12%)	4 (14%)	22 (79%)	2 (7%)
2020	26 (11%)	6 (23%)	17 (65%)	3 (12%)
2021	26 (11%)	11 (42%)	10 (38%)	5 (19%)
2022	34 (14%)	8 (24%)	15 (44%)	11 (32%)
*Country*				
United Kingdom	54 (22%)	16 (30%)	33 (61%)	5 (9%)
United States	37 (15%)	13 (35%)	21 (57%)	3 (8%)
China	33 (14%)	11 (33%)	10 (30%)	12 (36%)
Canada	9 (4%)	1 (11%)	7 (78%)	1 (11%)
Japan	6 (2%)	1 (17%)	3 (50%)	2 (33%)
Europe^a^	78 (32%)	12 (15%)	62 (79%)	4 (5%)
Latin America^b^	7 (3%)	2 (29%)	4 (57%)	1 (14%)
Other^c^	20 (8%)	4 (20%)	11 (55%)	5 (25%)
Unknown	2 (1%)	2 (100%)	0 (0%)	0 (0%)
*Study population*				
Type 2 diabetes	208 (86%)	47 (23%)	134 (64%)	27 (13%)
Type 1 diabetes	24 (10%)	1 (4%)	22 (92%)	1 (4%)
Diabetic macular edema	22 (9%)	14 (64%)	3 (14%)	5 (23%)
Diabetic retinopathy	1 (0.4%)	0 (0%)	1 (100%)	0 (0%)
*Study perspective*				
Health care payer	213 (88%)	54 (25%)	130 (61%)	29 (14%)
Societal	32 (13%)	8 (25%)	19 (59%)	5 (16%)
Private health care payer	1 (0.4%)	0 (0%)	1 (100%)	0 (0%)
Provider	1 (0.4%)	0 (0%)	0 (0%)	1 (100%)
Unknown	2 (1%)	0 (0%)	2 (100%)	0 (0%)
*Time horizon*				
≤1 year	19 (8%)	0 (0%)	19 (100%)	0 (0%)
>1 and <10 years	16 (7%)	3 (19%)	11 (69%)	2 (13%)
≥10 and <30 years	22 (9%)	10 (45%)	7 (32%)	5 (23%)
≥30 years	182 (75%)	47 (26%)	113 (62%)	22 (12%)
Unknown	5 (2%)	2 (40%)	0 (0%)	3 (60%)
*Model type*				
Simulation model	139 (57%)	32 (23%)	92 (66%)	15 (11%)
Markov model (state-transition model)	44 (18%)	14 (32%)	19 (43%)	11 (25%)
Microsimulation model	15 (6%)	8 (53%)	4 (27%)	3 (20%)
Semi-Markov model^d^	10 (4%)	3 (30%)	7 (70%)	0 (0%)
Discrete event simulation model	11 (5%)	3 (27%)	7 (64%)	1 (9%)
Decision tree	3 (1%)	1 (33%)	0 (0%)	2 (67%)
Partitioned survival model	1 (0.4%)	0 (0%)	1 (100%)	0 (0%)
No specification	19 (8%)	1 (5%)	18 (95%)	0 (0%)
*Model*				
Cardiff T1DM^273^	1 (0.4%)	1 (100%)	0 (0%)	0 (0%)
Cardiff T2DM^274^	28 (12%)	17 (61%)	11 (39%)	0 (0%)
IQVIA CORE Diabetes Model^275^ ^*e*^	91 (38%)	13 (14%)	75 (82%)	3 (3%)
United Kingdom Prospective Diabetes Study Outcomes Model 2 (UKPDS-OM2)^276^	15 (6%)	1 (7%)	7 (47%)	7 (47%)
Swedish Institute for Health Economics Cohort Model for T2DM (IHECM T2DM)^277^	11 (5%)	0 (0%)	10 (91%)	1 (9%)
Chinese Outcomes Model for T2DM (COMT)^278^	4 (2%)	1 (25%)	2 (50%)	1 (25%)
Economic and Health Outcomes Model of T2DM (ECHO-T2DM)^279^	4 (2%)	3 (75%)	1 (25%)	0 (0%)
The PRIME model^280^	1 (0.4%)	0 (0%)	1 (100%)	0 (0%)
Cardiff Research Consortium Discrete Event Simulation (CRC DES) model^43^	2 (1%)	0 (0%)	2 (100%)	0 (0%)
The Archimedes model^281^	1 (0.4%)	0 (0%)	0 (0%)	1 (100%)
Model by Kansal^139^	4 (2%)	1 (25%)	1 (25%)	2 (50%)
Model by Viriato^267^	2 (1%)	0 (0%)	2 (100%)	0 (0%)
Model by Ericsson^77^	10 (4%)	0 (0%)	10 (100%)	0 (0%)
Model by Ridderstråle^231^	3 (1%)	0 (0%)	3 (100%)	0 (0%)
Model by Valentine^260^	3 (1%)	0 (0%)	3 (100%)	0 (0%)
Model by Abushanab^31^	2 (0.8%)	0 (0%)	0 (0%)	2 (100%)
Model self-made by authors	50 (21%)	17 (34%)	20 (40%)	13 (26%)
Health technology appraisal conducted by NICE or CADTH	11 (5%)	8 (73%)	3 (27%)	0 (0%)
Model by NICE	2 (1%)	0 (0%)	0 (0%)	2 (100%)

NOTE: Values may not total 100% because some publications included several choices.

ADEs, adverse drug events; CADTH, Canadian Agency for Drugs and Technologies in Health; CEAs, cost-effectiveness analyses; NICE, National Institute for Health and Care Excellence; T1DM, type 1 diabetes mellitus; T2DM, type 2 diabetes mellitus.

^a^Europe: Austria, Bulgaria, Czech Republic, Denmark, Estonia, Finland, France, Germany, Greece, Italy, Netherlands, Norway, Poland, Portugal, Serbia, Slovakia, Spain, Sweden, Switzerland.

^b^Latin America: Argentina, Brazil, Colombia, Ecuador, Mexico.

^c^Other countries: Algeria, Australia, Hong Kong, India, Indonesia, Iran, Malaysia, Saudi Arabia, Singapore, South Korea, Taiwan, Thailand, Vietnam, Qatar.

^d^Semi-Markov: Elements of Markov model and other types of models.

^e^Also termed the IMS CORE Diabetes Model.

### Review findings

The review findings are presented according to the concept of this scoping review. Firstly, inclusion of ADEs in the cost-effectiveness analyses is examined overall and in terms of modeling type and the disease. Secondly, the question of how ADEs are incorporated in the cost-effectiveness analyses is examined according to disease.

#### Inclusion of adverse drug events in the cost-effectiveness analyses

As presented in Table 1, 26% (n=62) of the included cost-effectiveness analyses included ADEs in the modeling, and 13% (n=32) did not. Most of the analyses (61%; n=148) partly considered ADEs; that is, the analyses included 1 or 2 ADEs but omitted many other relevant ones reported in efficacy studies on which the model was based on and/or in the summary of product characteristics. Usually, partial inclusion meant taking only hypoglycemia or only hypoglycemia and weight gain into account (78% and 90% of the “partly” category, respectively), but excluding all other ADEs. Hypoglycemia is not straightforward in terms of terminology; some of the included analyses reported hypoglycemia as an ADE, and some reported it as a complication of diabetes. Because pharmaceutical interventions often include hypoglycemia as an ADE in their summaries of product characteristics, a wider definition was deemed appropriate, and hypoglycemia was considered as an ADE in this review. If hypoglycemia had not been defined as an ADE, the number of cost-effectiveness analyses partly including ADEs and the number excluding ADEs would be reversed (ie, 13% of analyses would have partly included ADEs and 61% would have excluded ADEs).

The year of publication showed a negligible correlation to the inclusion of ADEs, although a slight trend was observed with more recent publications excluding ADEs more often than older publications. This trend did not exist for models that either completely or partly included ADEs in the model. The perspective of the analysis (eg, health care or societal) did not show a trend with the inclusion of ADEs. The analyses with longer time horizons (>10 years) better included ADEs than the analyses with shorter time horizons, although the analyses with longer time horizons also excluded ADEs more often than the those with shorter time horizons. Of the model types, microsimulation models had the largest proportion of analyses including ADEs (53%; n=8), whereas the decision trees had the largest proportion of analysis excluding ADEs (67%; n = 2).

In general, NICE and CADTH evaluations captured ADEs well (73%; n=8). A considerable proportion of analyses using the ECHO-T2DM and Cardiff-T2DM models included ADEs (75% [n=3] and 61% [n=17], respectively). On the other hand, a notable proportion of analyses using UKPDS-OM2 excluded ADEs (47%; n=7). With the self-made models, inclusion of ADEs was fairly evenly distributed to full inclusion (34%; n=17), part inclusion (40%; n=20), and exclusion (26%; n=13). The analyses of the other individual models included ADEs in varying proportions.

The inclusion of ADEs also varied between diseases (see Table 2). Of the models for T1DM, only 4% (n=1) included ADEs, whereas 23% (n=47) of the models for T2DM and 61% (n=14) of the models for DR and DME (n=23) included ADEs. A majority of the models that incorporated ADEs included them in the cost estimates (T1DM: 100% [n=1]; T2DM: 72% [n=34]; DR and DME: 100% [n= 14]). Additionally, most T1DM and T2DM models that incorporated ADEs included them in the quality-of-life estimates (T1DM: 100% [n=1]; T2DM: 89% [n=42]), but for DR and DME models, the proportion was considerably lower (43%; n = 6).

**Table 2:** Incidence of inclusion of adverse drug events in cost-effectiveness analyses of pharmacological interventions, by condition (type 1 diabetes mellitus, type 2 diabetes mellitus, and diabetic retinopathy/diabetic macular edema)

Incidence of ADEs included	Type 1 diabetes mellitus (n = 24)	Type 2 diabetes mellitus (n = 208)	Diabetic retinopathy and diabetic macular edema (n = 23)
**Yes (included)**	**1 (4%)**	**47 (23%)**	**14 (61%)**
Included ADEs	Hypoglycemia (100%), diabetic ketoacidosis (100%), urinary tract infection (100%)	Hypoglycemia (94%), genital infection (68%), urinary tract infection (60%), weight gain (47%), nausea (21%), gastrointestinal events (13%), ketoacidosis (13%), diabetic ketoacidosis (6%), vomiting (6%), injection reaction (6%), acute kidney injury (6%), fractures (4%), volume depletion (4%), osmotic diuresis (4%), lower limb amputation (2%), lactic acidosis (2%), headache (2%), upper respiratory tract infection (2%), nasopharyngitis (2%), diarrhea (2%), dizziness (2%), hypotension (2%)	Cataract (86%), endophthalmitis (71%), retinal detachment (71%), glaucoma (50%), intraocular pressure rise (50%), vitreous hemorrhage (50%), myocardial infarction (14%), thromboembolic events (14%), vitrectomy (14%), cerebrovascular accident (7%), lens damage (7%), ocular inflammation (7%)
*ADEs included in the cost estimates*			
Yes	1 (100%)	34 (72%)	14 (100%)
Partly	0 (0%)	10 (21%)	0 (0%)
No	0 (0%)	1 (2%)	0 (0%)
Unclear	0 (0%)	2 (4%)	0 (0%)
*ADEs included in the QoL estimates*			
Yes	1 (100%)	42 (89%)	6 (43%)
Partly	0 (0%)	3 (6%)	1 (7%)
No	0 (0%)	1 (2%)	7 (50%)
Unclear	0 (0%)	1 (2%)	0 (0%)
*ADEs included in the cost and the QoL estimates*			
Yes: costs, Yes: QoL	1 (100%)	30 (64%)	6 (43%)
Yes: costs, Partly: QoL	0 (0%)	2 (4%)	1 (7%)
Yes: costs, No: QoL	0 (0%)	1 (2%)	7 (50%)
Yes: costs, Unclear: QoL	0 (0%)	1 (2%)	0 (0%)
Partly: costs, Yes: QoL	0 (0%)	9 (19%)	0 (0%)
Partly: costs, Partly: QoL	0 (0%)	1 (2%)	0 (0%)
Partly: costs, No: QoL	0 (0%)	0 (0%)	0 (0%)
Partly: costs, Unclear: QoL	0 (0%)	0 (0%)	0 (0%)
No: costs, Yes: QoL	0 (0%)	1 (2%)	0 (0%)
No: costs, Partly: QoL	0 (0%)	0 (0%)	0 (0%)
No: costs, No: QoL	0 (0%)	0 (0%)	0 (0%)
No: costs, Unclear: QoL	0 (0%)	0 (0%)	0 (0%)
Unclear: costs, Yes: QoL	0 (0%)	2 (4%)	0 (0%)
Unclear: costs, Partly: QoL	0 (0%)	0 (0%)	0 (0%)
Unclear: costs, No: QoL	0 (0%)	0 (0%)	0 (0%)
Unclear: costs, Unclear: QoL	0 (0%)	0 (0%)	0 (0%)
**Partly included**	**22 (92%)**	**134 (64%)**	**4 (17%)**
*ADEs included in the cost estimates*			
Yes	20 (91%)	99 (74%)	4 (100%)
Partly	1 (5%)	17 (13%)	0 (0%)
No	1 (5%)	8 (6%)	0 (0%)
Unclear	0 (0%)	10 (7%)	0 (0%)
*ADEs included in the QoL estimates*			
Yes	19 (86%)	113 (84%)	0 (0%)
Partly	2 (9%)	4 (3%)	0 (0%)
No	0 (0%)	7 (5%)	4 (100%)
Unclear	1 (5%)	10 (7%)	0 (0%)
*ADEs included in the cost and the QoL estimates*			
Yes: costs, Yes: QoL	18 (82%)	91 (68%)	0 (0%)
Yes: costs, Partly: QoL	1 (5%)	2 (1%)	0 (0%)
Yes: costs, No: QoL	0 (0%)	4 (3%)	4 (100%)
Yes: costs, Unclear: QoL	1 (5%)	2 (1%)	0 (0%)
Partly: costs, Yes: QoL	0 (0%)	13 (10%)	0 (0%)
Partly: costs, Partly: QoL	1 (5%)	2 (1%)	0 (0%)
Partly: costs, No: QoL	0 (0%)	1 (1%)	0 (0%)
Partly: costs, Unclear: QoL	0 (0%)	1 (1%)	0 (0%)
No: costs, Yes: QoL	1 (5%)	6 (4%)	0 (0%)
No: costs, Partly: QoL	0 (0%)	0 (0%)	0 (0%)
No: costs, No: QoL	0 (0%)	2 (1%)	0 (0%)
No: costs, Unclear: QoL	0 (0%)	0 (0%)	0 (0%)
Unclear: costs, Yes: QoL	0 (0%)	3 (2%)	0 (0%)
Unclear: costs, Partly: QoL	0 (0%)	0 (0%)	0 (0%)
Unclear: costs, No: QoL	0 (0%)	0 (0%)	0 (0%)
Unclear: costs, Unclear: QoL	0 (0%)	7 (5%)	0 (0%)
**No (not included)**	**1 (4%)**	**27 (13%)**	**5 (22%)**
*ADEs reported in the source of efficacy results*			
Yes	0 (0%)	19 (70%)	5 (100%)
No	0 (0%)	4 (15%)	0 (0%)
Unclear	1 (100%)	4 (15%)	0 (0%)
Justification provided for not including ADEs	0 (0%)	6 (26%)	5 (100%)

ADEs, adverse drug events; QoL, quality of life.

The partial inclusion of ADEs was more commonly reported in the models for T1DM (92%; n=22) and T2DM (64%; n=134). Regarding models for DR and DME, partial inclusion existed in 17% (n=4) of the models. The models that partially included ADEs captured them well in the cost estimates (T1DM: 91% [n=20]; T2DM: 74% [n=99]; DR and DME: 100% [n=4]), but some variability existed with regard to inclusion in quality-of-life estimates (T1DM: 86% [n=19]; T2DM: 84% [n=113]; DR and DME: 0%).

#### How adverse drug events were incorporated in the cost-effectiveness analyses

Not all models that included ADEs used them in both cost and quality-of-life estimates. Of the models that fully included ADEs in the analysis, 100% (n=1) of the T1DM models, 64% (n=30) of the T2DM models, and 43% (n=6) of the DR or DME models included ADEs in both costs and quality-of-life estimates (Table 2). Similarly, of the models that partly included ADEs in the analysis, 68% (n=91) of T2DM models included the impact of ADEs on both costs and quality of life, and none of the DR or DME models that partly included ADEs in the analysis included ADEs in both cost and quality-of-life estimates. With regard to models for DR and DME, ADE-related costs appear to have been well incorporated, but the ADE-related impact on quality of life seems to be less well considered.

The ADEs were completely excluded from 4% (n = 1) of the T1DM models, 13% (n=27) of the T2DM models, and 22% (n=5) of the DR and DME models, although the majority of the efficacy sources for the models reported ADEs. The analyses provided justification for exclusion in a varying manner; for example, only 26% (n=6) of the analyses for T2DM provided justification, whereas all 5 (100%) analyses for DR and DME provided justification. The reasons for excluding ADE included low incidence of ADEs, no (statistical) difference in incidence between comparators, assumed negligible impact, no impact on long-term quality of life, and ADEs being a transient event.

The most common ADEs that were included for T1DM and T2DM models were hypoglycemia, diabetic ketoacidosis, urinary tract infection, genital infection, weight gain, nausea, and gastrointestinal events. The most common ADEs for models for DR and DME included cataract, endophthalmitis, retinal detachment, glaucoma, intraocular pressure rise, and vitreous hemorrhage.

Table 3 presents the data sources used for ADE incidences, costs, and disutility measures. Table 3 only considers the analyses that either completely or partly included ADEs. Overall, the most common source of ADE incidences were clinical trials (65% of the analyses; n = 136), followed by meta-analyses (14%; n=30), observational studies (13%; n=28), and network meta-analyses (11%; n = 23). The most notable difference between different conditions was that analyses for DME and DR based their ADE incidences on clinical trials (89%; n=16), whereas analyses for T1DM and T2DM more broadly considered different data sources for ADE incidences. The most common sources of ADE costs were published (peer-reviewed) literature (60%; n=119) and unit costs/databases (39%; n=77). Compared with the analyses for T1DM and T2DM, the analyses for DR and DME more often used unit costs/databases (83%; n=15) and expert opinions (11%; n=2) as the sources of ADE costs. With regard to ADE disutility measures, EQ-5D was the most used measure (47%; n=90) followed by time trade-off (TTO; 46%; n=87). Compared with the analyses for T1DM and T2DM, the analyses for DR and DME more often included assumption as a source of disutility (14%; n=1), or the source for the disutility remained unclear (57%; n = 4).

**Table 3:** Data sources of adverse drug events in cost-effectiveness analyses of pharmacological interventions, by condition (type 1 diabetes mellitus, type 2 diabetes mellitus, and diabetic retinopathy/diabetic macular edema)

Data sources	Type 1 diabetes mellitus (n = 23)	Type 2 diabetes mellitus (n = 181)	Diabetic retinopathy and diabetic macular edema (n = 18)	All (n = 210)
*Source of ADE incidences*				
Clinical trial	8 (35%)	114 (63%)	16 (89%)	136 (65%)
Meta-analysis	5 (22%)	28 (15%)	0 (0%)	30 (14%)
Observational study	11 (48%)	24 (13%)	2 (11%)	28 (13%)
Network meta-analysis	1 (4%)	20 (11%)	2 (11%)	23 (11%)
Indirect treatment comparison	0 (0%)	15 (8%)	1 (6%)	16 (8%)
Previous economic evaluation	0 (0%)	2 (1%)	0 (0%)	2 (1%)
Expert opinion	0 (0%)	1 (1%)	0 (0%)	1 (0.5%)
Unknown	0 (0%)	1 (1%)	0 (0%)	1 (0.5%)
*Source of ADE cost estimates* ^*a*^				
Published literature^b^	16 (73%)	110 (69%)	1 (6%)	119 (60%)
Unit costs from national/hospital/local database^c^	7 (32%)	58 (36%)	15 (83%)	77 (39%)
Expert opinion	0 (0%)	3 (2%)	2 (11%)	5 (3%)
Assumption	0 (0%)	3 (2%)	0 (0%)	3 (2%)
Unclear	0 (0%)	9 (6%)	2 (11%)	11 (6%)
*PROM measure/method for disutilities* ^*a*^				
EQ-5D	7 (32%)	80 (49%)	3 (43%)	90 (47%)
Time trade-off, TTO	16 (73%)	81 (50%)	1 (14%)	87 (46%)
Standard gamble	1 (5%)	18 (11%)	0 (0%)	19 (10%)
The Index of Well-Being	1 (5%)	12 (7%)	0 (0%)	13 (7%)
Health Utility Index	0 (0%)	5 (3%)	0 (0%)	5 (3%)
Assumption	0 (0%)	1 (1%)	1 (14%)	2 (1%)
Visual Function Questionnaire-25	0 (0%)	0 (0%)	1 (14%)	1 (1%)
Unclear	1 (5%)	20 (12%)	4 (57%)	25 (13%)

ADE, adverse drug event; PROM, patient-reported outcome measure

^a^Analyses that either completely or partly included costs are considered in the table. An analysis may include multiple options.

^b^Peer-reviewed publication.

^c^Including National Health Service reference costs, claims data, and Medicare.

Appendix V contains more condition-specific information on the models with regard to modeling method and general PROM measures used for health state utilities. Additionally, Table 4 presents per condition how ADEs were included in the model. The analyses included ADEs in the models as probabilities/incidence rates (55%; n=123), a submodel (40%; n=88), a risk equation (3%; n=7), or a separate health state (2%; n=4). The efficacy sources in the model often reported the incidence of ADEs as per 100 or 1000 patient-years. The models for DR and DME only included ADEs as probabilities, whereas models for T1DM and T2DM had more variability in the inclusion of ADEs.

**Table 4:** Modeling method for adverse drug events in cost-effectiveness models of pharmacological interventions, by condition (type 1 diabetes mellitus, type 2 diabetes mellitus, and diabetic retinopathy/diabetic macular edema)

How adverse drug events were included^a^	Type 1 diabetes mellitus (n = 23)	Type 2 diabetes mellitus (n = 181)	Diabetic retinopathy and diabetic macular edema (n = 18)	All (n = 222)
Probabilities	16 (70%)	99 (55%)	18 (100%)	123 (55%)
Submodel	7 (30%)	81 (45%)	0 (0%)	88 (40%)
Risk equation	0 (0%)	7 (4%)	0 (0%)	7 (3%)
Separate health state	0 (0%)	4 (2%)	0 (0%)	4 (2%)

^a^Analysis may include multiple options (eg, submodel for one adverse drug event and probability for another).

The disutilities related to ADEs were calculated by multiplying the proportion of patients who experienced the event by the expected disutility related to the specific event. This was also weighted with the duration of disutility. Generally, disutilities were applied in the year the ADE occurred and for each subsequent year, when appropriate. Some analyses (eg, Gu et al.^108^ and Ishii et al.^134^) did not apply disutility for subsequent years in order to reflect the transient nature of ADEs. Disutilities for ADEs were additive, meaning that a patient experiencing multiple events in a given year would also receive all corresponding disutilities for that year. For the costs related to ADEs, the calculation simply included multiplying the cost per event by the incidence of such an ADE. Similar principles as with disutilities (eg, application for 2 year and sometimes beyond, multiple events accounted with additive manner) applied to ADE-related costs.

Discontinuation due to ADEs was accounted for in some of the models. Many of the models that included hypoglycemia as an ADE applied a diminishing marginal utility model^282^ in their base case or in the sensitivity analyses. This approach considers a decreasing impact of each subsequent event on quality of life based on a log-transformed regression equation.

The ADEs’ impact on the results of cost-effectiveness analysis varied between the models. In some models, the impact of ADEs on the cost-effectiveness analysis results was negligible (eg, Perez *et al.* ^202^ and Raya *et al.* ^226^). In some models, the impact of ADEs was deemed significant (eg, Lalic *et al.*,^146^ Lin *et al.*,^154^ Morales *et al.* ^178^) or minor (eg, Reifsnider *et al.* ^230^ and Zupa *et al.* ^272^). The diminishing marginal utility model in general seemed to have little impact on the results of analyses. Other discussion topics in the cost-effectiveness models included challenges in treatment adherence due to ADEs, productivity losses and other indirect costs due to ADEs, and the difference of clinical trials and routine clinical practice in detecting relevant ADEs.

### Discussion

ADEs are a relevant outcome to consider when modeling cost-effectiveness, yet the guidelines for economic evaluation seem vague with regard to ADEs, and the practices for incorporating ADEs in economic evaluations vary. This scoping review examined a great number of published economic evaluations for T1DM, T2DM, DR, and DME. According to the results, only 26% (n=62) of the included cost-effectiveness models considered ADEs, and 13% (n=32) of the models completely excluded them. The definition of hypoglycemia as an ADE considerably impacted the results of this review; if hypoglycemia were considered a complication of a disease rather than an ADE, up to 61% of the models would have excluded ADEs completely. The results were in line with other reviews examining inclusion of ADEs in economic models^12,13,24^ in which inclusion of ADEs was considered varying and incomplete. In this review, most models with a short time horizon (<10 years) partly included ADEs in the analysis, whereas models with longer time horizon more often varied in terms of level of inclusion. The impact of time horizon on the inclusion of ADEs in the model, therefore, was not as straightforward as reported in the review by Craig *et al.*,^12^ which found a trend of models with longer time horizons better capturing ADEs compared to models with shorter time horizons.

Diabetes (types 1 and 2) and its ocular complications, DR and DME, were chosen for this review to enable comparison of economic models for different conditions with different amounts of existing literature. As a hypothesis, models for diabetes were thought to better include ADEs because research into diabetes is broader and more extensive. According to the results of this review, however, there was not a large difference in inclusion of the incidence of ADEs in economic models between the conditions. A larger percentage of the models for DME and DR fully considered the incidence of ADEs compared with the models for T1DM and T2DM, but also more often excluded ADEs from the model entirely. Models for T1DM and T2DM, in general, partly considered ADEs, but usually only for hypoglycemia. If hypoglycemia had not been considered an ADE, then the models for diabetes would have captured ADEs more poorly than the models for DME and DR.

Some condition-related differences existed between the models that included ADEs. Regarding costs, the models for DME and DR always included ADE-related costs, but models for diabetes had slightly more variation. More prevalent difference between the conditions existed in terms of including ADEs in the quality-of-life estimates for the modeling. Half (n=7) of the models for DR and DME excluded the impact of ADEs on quality of life, whereas only 2% (n=1) of the models for diabetes excluded ADE-related impact on quality of life. For the models that partly included ADEs in the modeling, a similar pattern was observed; models for DME and DR better included ADE-related costs in the modeling but completely excluded ADE-related impact on quality of life.

It is possible that the models could include ADEs only as quality-of-life parameters and not in cost parameters, interpreted as ADEs having a clinical impact but no impact on health care resource use. Inclusion of ADEs in quality-of-life parameters only and not in cost parameters was rare for the models included in this scoping review and occurred only in models for T2DM. Another situation in which ADEs were included in cost parameters and not in quality-of-life parameters was more common in the included models. This was visible for the models for DR and DME in which the impact of ADEs on quality of life was often excluded, although ADE-related costs were always included. One potential explanation for the exclusion of the impact of ADEs on quality of life could be that the models for ocular conditions were considered to already capture ADEs in the vision acuity–based health states, thus additional disutilities would have caused double counting of ADEs.

More than half of the models (64%) for T1DM and T2DM that incorporated ADEs included them in both cost and quality-of-life estimates, whereas less than half (43%) of the models for DR and DME included ADEs in both cost and quality-of-life estimates. These results align with a previously published review that tracked the inclusion of ADEs in health technology assessment reports and found that 60% of the reports considered ADEs in estimates for both costs and effectiveness, and of these parameters, ADE-related costs were better captured than ADE-related impact on effectiveness.^12^ In addition, the inclusion rate of ADE-related disutilities in economic models for cancer treatments has been reported to be 54%,^13^ which, based on the results of this scoping review, is less than that of the models for T1DM and T2DM (100% and 89%, respectively) but more than in the models for DR and DME (43%; see Table 2).

Based on the information collected in this scoping review, some potential explanations for the difference of inclusion of ADE-related disutilities across the conditions could be related to modeling methods. The models for T1DM and T2DM included ADEs as a submodel as well as probabilities, whereas models for DR and DME only included ADEs as probabilities (see Table 4). The model structure that already has a predefined health state for ADEs may better encourage the inclusion of ADE-related impact on quality of life. Additionally, as presented in Appendix V, the majority of models for DR and DME were Markov state-transition models, whereas for T1DM and T2DM, other types of modeling methods (eg, simulation models) were more common. The models for DR and DME were usually self-made by authors, whereas the models for T1DM and T2DM were validated, vastly researched models. The use of more established, validated models may increase the likelihood of accounting for ADE-related costs and disutilities in the analysis. However, this was not observable in the inclusion of ADEs in the models in general, as the models for T1DM and T2DM did not show better overall inclusion of the incidence of ADEs compared with models for DR and DME.

The difference between diabetes models and models for DR and DME in inclusion of ADE-related impact on quality of life may also be related to measures used for estimating health-related quality of life. The most common quality-of-life measure was the EQ-5D for the T1DM and T2DM models, whereas the time trade-off (TTO) method was more common for the DR and DME models (Appendix V). The quality-of-life measure for disutilities was more often unclear for models in DR and DME than for models in T1DM and T2DM (see Table 3).

Some challenges regarding the use of the TTO method in valuating temporary health states, such as ADEs, have been identified, primarily related to the evaluation of utilities for short-term health states as if they were long-term health states being constant over time. The typical TTO method, therefore, could bias the utility estimation for temporary health states.^283^ The challenges related to the TTO method may not explain why models for DR and DME inadequately considered ADE-related impact on quality of life, but it could indicate that even the models that did consider ADE-related impact on quality of life did not necessarily capture their real impact when using the TTO method for utility elicitation.

Several reasons for excluding ADEs from the models existed. For example, Evans *et al.* ^87^ only applied ADEs to the model when there was a statistical difference in ADEs between the treatments. Also, Morales *et al.* ^178^ only accounted for statistically significant clinical outcomes, and Lasalvia *et al.* ^149^ assumed equal rates for ADEs when there was no statistical difference between treatments. This justification was also included in an evaluation conducted by NICE^193^ in addition to excluding ADEs with low incidence. Luo *et al.* ^158^ also excluded rare ADEs, and Reifsnider *et al.* ^228,230^ modeled only ADEs that were reported in at least 5% of patients using the treatments. Torre *et al.* ^254^ excluded mild ADEs because of their negligible economic impact.

The reasons for excluding ADEs were well in line with the reasons identified in existing literature. For example, Lu *et al.* ^13^ identified that ADEs that did not differ in their incidences between the treatments, or ADEs that were rare and/or mild, were often excluded from economic models. Additionally, the authors concluded that lack of (disutility) data could explain exclusion of the impact of ADEs in the models.^13^ In this scoping review, the most common source of ADE incidence was clinical trials, and it was often discussed in the included publications whether clinical trials were good enough data for capturing all the relevant ADEs. ADEs are a demanding outcome to quantify and valuate; the short follow-up period of randomized clinical trials may not capture ADEs occurring in later stages of the treatment, and observational studies usually lack causality between an ADE and the treatment. In addition, valuation of many mild ADEs compared with a single severe ADE could be challenging.

Sometimes ADEs are excluded from the models because of an assumption that ADEs are already considered in the health state utilities. For example, Craig *et al.* ^12^ concluded that the models that included utilities elicited directly from the patients receiving treatment already accounted for the impact of ADEs. The risk of double counting of ADEs is a potential challenge for the models in situations where health state utilities are directly elicited from the population in which ADEs may also occur during the follow-up period and additional disutility for ADEs has still been incorporated in the model.^15^ This topic would need more research to better understand its implications. Regarding the models included in this scoping review, the potential double counting of ADEs was only mentioned in a few included publications, and thus it was not an explanatory factor for excluding ADEs from the models.

### Limitations and strengths

One strength of this scoping review was the modification of the a priori protocol^27^ to achieve reliable results for this review, mainly related to the types of sources eligible for inclusion in this scoping review. Another strength was that the data extraction form was modified to better serve the needs of the data analysis for this scoping review.

The limitations of this scoping review included the exclusion of languages other than English, and the time between the literature search and the completion of the review. Regarding the gray literature, only the health technology assessment databases for NICE and NHS were considered in this scoping review. The search also located some evaluations by CADTH that were included in the sample of studies; EUnetHTA provided no evaluations for this review. According to the guidelines for systematic reviews of economic evaluations, NICE is a recommended database to consider, CADTH is optional, and other databases have not been mentioned.^283^ In that sense, the included studies in this scoping review are also in line with the guidelines for a review of economic evaluations.

Not all information in the data extraction form was available in the publications, leading to the use of “uncertain” categories when extracting the data. The use of these categories was occasional, so it should not have affected the results in this scoping review. The categorization of the model types (eg, Markov model, simulation model, microsimulation model) was based on terminology used in the included articles. Typically, a simulation model is used for modeling individual patients and the Markov model for modeling a cohort, but because Markov models also enable patient-level simulation, it is possible that some of the Markov models are simulation models as well.

Regarding the content of the review, more focus could have been placed on capturing ADE-related discontinuations in the models. The current data extraction form excluded this topic but enabled addressing the topic in free text sections. The free text, however, is not a structured way of collecting this information. According to the information collected from the free text sections, only 9% of the included models considered ADE-related discontinuation. Compared with findings from Craig *et al.* ^12^ in which 30% of the analyses reported ADE-related discontinuation, some observations in this scoping review regarding this topic were most likely unnoticed. This information could have been relevant to capture, as discontinuation of treatment in clinical studies stops the follow-up of patients, thus excluding those who experienced ADEs from the overall population. This leads to underestimation of ADEs in the overall study population.

## Conclusions

Based on the results of this scoping review, the inclusion of ADEs into cost-effectiveness models for pharmacological treatments for T1DM, T2DM, DR, and DME is suboptimal. Not interpreting hypoglycemia as an ADE notably affected the results, showing a poor inclusion of ADEs in cost-effectiveness models in general. The ADE-related costs were better captured than the ADE-related impact on quality of life, and this was especially shown in models for DR and DME. The most common source of ADEs were clinical trials, and most often ADEs were incorporated as probabilities to the models.

Not all models that excluded ADEs provided justification for it, and the ones that did had many reasons for the exclusion. The common issue regarding ADEs in economic evaluation seems to be disregard and inconsistent consideration of ADEs in cost-effectiveness models. It could be useful for guidelines of economic evaluation to more clearly encourage inclusion of ADEs and to provide better technical instruction on how to include them. This could standardize the inclusion of ADEs in cost-effectiveness models. In order to have all the relevant evidence considered in economic evaluation, including ADEs in cost-effectiveness models should be a fundamental part of it.

### Implications for research

This scoping review has disclosed inconsistent inclusion of ADEs in economic evaluation of pharmacological interventions used for the treatment of T1DM and T2DM, DR, and DME. Although economic evaluation aims to incorporate all the relevant evidence in the analysis, ADEs do not receive proper attention in guidelines or practical execution of economic evaluation. Future research should more closely investigate the potential impact of ADEs on the results, and identify the criteria and policies for practical inclusion of ADEs in economic evaluation. Better recognition of ADEs would ensure that the information provided by economic evaluation is comprehensive and adequately captures the relevant outcomes of the interventions in comparison.^184^

## Acknowledgments

Maarit Putous for her help with defining the search strategy and conducting the search.

This review contributes toward a degree award (PhD) for MP.

## Funding

MP has received funding from Evald ja Hilda Nissi Foundation and Sokeain Ystävät ry. The funders had no role in the review process.

## Author contributions

MP, EK, and VJ wrote the a priori protocol. MP and EK/VJ screened and did full-text review for the records in this review. MP did the data extraction for all included studies, and EK verified the work by conducting data extraction for 20% of the included studies. MP did the data analysis and wrote the manuscript, having responsibility for the final content of the manuscript. EK wrote and edited the manuscript, and both EK and VJ advised on the methodology and the content. MP, EK, and VJ read and approved the final review.

## Availability of data, code, and other materials

An a priori protocol is published open access. Search strategy, studies ineligible following full-text review, and data extraction instrument are included in the appendices of this review. The full data extraction is available as supplemental content: http://links.lww.com/SRX/A52.

## Supplementary Material

## Appendix I: Search strategy

### MEDLINE (Ovid)

Search conducted: January 1, 2023

**Table:**

Query	Records retrieved
(“Diabetes mellitus” [MeSH Terms] OR “Diabetes” [Title/Abstract] OR “Diabetic retinopathy” [MeSH Terms] OR “Diabetic retinopathy” [Title/Abstract] OR “Diabetic macular oedema” [Title/Abstract] OR “Diabetic macular edema” [Title/Abstract]) AND (“Cost-effectiveness” [Title/Abstract] OR “Cost-utility” [Title/Abstract] OR “Economic evaluation” [Title/ Abstract] OR “Economic model” [Title/Abstract] OR “Markov*” [Title/Abstract])	2910
Limited to 1.1.2011–31.12.2022, English, Full text	

### Scopus

Search conducted: January 1, 2023

**Table:**

Query	Records retrieved
((TITLE-ABS-KEY (diabetes OR “diabetes mellitus” OR “diabetic retinopathy” OR “diabetic macular edema” OR “diabetic macular oedema”)) AND (TITLE-ABS-KEY (“cost-effectiveness” OR “cost-utility” OR “economic evaluation” OR “economic model*“ OR markov))) AND (LIMIT-TO (SRCTYPE, “j”)) AND (LIMIT-TO (LANGUAGE, “english”)) AND (LIMIT-TO (DOCTYPE, “ar”) OR LIMIT-TO (DOCTYPE, “re”)) AND (LIMIT-TO (SUBJAREA, “medi”) OR LIMIT-TO (SUBJAREA, “phar”)) AND (LIMIT-TO (PUBYEAR, 2022) OR LIMIT-TO (PUBYEAR, 2021) OR LIMIT-TO (PUBYEAR, 2020) OR LIMIT-TO (PUBYEAR, 2019) OR LIMIT-TO (PUBYEAR, 2018 ) OR LIMIT-TO (PUBYEAR, 2017) OR LIMIT-TO (PUBYEAR, 2016) OR LIMIT-TO (PUBYEAR, 2015) OR LIMIT-TO (PUBYEAR, 2014) OR LIMIT-TO (PUBYEAR, 2013) OR LIMIT-TO (PUBYEAR, 2012) OR LIMIT-TO (PUBYEAR, 2011))	5074

### CINAHL (EBSCOhost)

Search conducted: January 1, 2023

**Table:**

Query	Records retrieved
((TI diabetes OR “diabetes mellitus” OR “diabetic retinopathy” OR “diabetic macular oedema” OR “diabetic macular edema”) OR (AB diabetes OR “diabetes mellitus” OR “diabetic retinopathy” OR “diabetic macular oedema” OR “diabetic macular edema”)) AND ((TI “cost-effectiveness” OR “cost-utility” OR “economic evaluation” OR “economic model*” OR Markov) OR (AB “cost-effectiveness” OR “cost-utility” OR “economic evaluation” OR “economic model*” OR Markov))	1241
Limited to 1.1.2011–31.12.2022, English	

### Web of Science Core Collection

Search conducted: January 1, 2023

**Table:**

Query	Records retrieved
(diabetes OR “diabetes mellitus” OR “diabetic retinopathy” OR “diabetic macular edema” OR “diabetic macular oedema” (Topic) and English (Languages) and 2022 or 2021 or 2020 or 2019 or 2018 or 2017 or 2016 or 2015 or 2014 or 2013 or 2012 or 2011 (Publication Years)) AND (“cost-effectiveness” OR “cost-utility” OR “economic evaluation” OR “economic model*” OR Markov (Topic) and 2022 or 2021 or 2020 or 2019 or 2018 or 2017 or 2016 or 2015 or 2014 or 2013 or 2012 or 2011 (Publication Years) and English (Languages)	3579
Limited to 1.1.2011–31.12.2022, English	

### International Network of Agencies for Health Technology Assessment

Search conducted: January 1, 2023

**Table:**

Query	Records retrieved
(“cost-effectiveness” OR “cost-utility” OR “economic evaluation” OR “economic model*“ OR markov)[Title/abs] AND (diabetes OR “diabetes mellitus” OR “diabetic retinopathy” OR “diabetic macular edema” OR “diabetic macular oedema”)[Title/abs] FROM 2011 TO 2022	132
Limited to 1.1.2011–31.12.2022, English	

### National Institute for Health and Care Research (NHS)

Search conducted: January 1, 2023

**Table:**

Query	Records retrieved
(“cost-effectiveness” OR “cost-utility” OR “economic evaluation” OR “economic model*“ OR markov)[Title/abs] AND (diabetes OR “diabetes mellitus” OR “diabetic retinopathy” OR “diabetic macular edema” OR “diabetic macular oedema”)[Any field]	18
Limited to 1.1.2011–31.12.2022	

### National Institute for Health and Care Excellence

Search conducted: January 1, 2023

**Table:**

Query*	Records retrieved
(diabetes OR “diabetes mellitus” OR “diabetic retinopathy” OR “diabetic macular edema” OR “diabetic macular oedema”) [Any field]	37
Limited to 1.1.2011–31.12.2022.	

*Query (“cost-effectiveness” OR “cost-utility” OR “economic evaluation” OR “economic model*“ OR markov)[Title/abs] AND (diabetes OR “diabetes mellitus” OR “diabetic retinopathy” OR “diabetic macular edema” OR “diabetic macular oedema”) provided no results, and therefore, the search was conducted based on included diseases.

### NHS Economic Evaluation Database

Search conducted: January 1, 2023

**Table:**

Query*	Records retrieved
(“cost-effectiveness” OR “cost-utility” OR “economic evaluation” OR “economic model*“ OR markov)[Title/abs] AND (diabetes OR “diabetes mellitus” OR “diabetic retinopathy” OR “diabetic macular edema” OR “diabetic macular oedema”)[any field]	16
Limited to 1.1.2011–31.12.2022.	

### European Network for Health Technology Assessment (EUnetHTA)

Search conducted: January 1, 2023

**Table:**

Query	Records retrieved
Assessments Archive (2006 – 2021)	No cost-effectiveness analyses matching the inclusion criteria
Limited to 1.1.2011–31.12.2022.	

## Appendix II: Studies ineligible following full-text review

**Table:**

Reason for exclusion	Number of records excluded (n=133)	Explanation
Only abstract available	59	Congress abstracts with no full text available
Ineligible outcomes	15	No quality-adjusted life years reported and/or no incremental cost-effectiveness ratio reported; excluded as per inclusion and exclusion criteria
Comparison of dosing/route of administration	12	Comparison of dosing schemes or route of administration rather than a comparison of different treatment interventions (drugs); excluded as per inclusion and exclusion criteria
Ineligible study design	9	Study design does not include modeling; excluded as per protocol
Duplicate	9	Duplicate
Review article^a^	7	(Systematic) literature reviews as per protocol were excluded
Language not English	6	Excluded as per protocol
Model validation study	5	A model validation study, not a cost-effectiveness analysis with intended comparison of interventions
Ineligible intervention	3	Intervention is not indicated for conditions included in the scoping review; excluded as per protocol
Ineligible indication	2	Indication not included in the review as per protocol
Editorial/commentary/summary	2	Summary type of article without comprehensive description of the included analyses
Health technology assessment report without (sufficient) modeling description	2	Health technology assessment report with confidential information; modeling insufficiently described
Protocol	1	A protocol, not a full analysis
Poster	1	A poster, not full-text article

^**a**^Excluded review articles hand-searched for additional applicable analyses.

### List of excluded sources

**Table:**

Citation details of excluded reports	Reason for exclusion
Abad Paniagua EJ, Casado Escribano P, Fernández Rodriguez JM, *et al.* Cost-effectiveness analysis of dapagliflozin compared to DPP4 inhibitors and other oral antidiabetic drugs in the treatment of type-2 diabetes mellitus in Spain. Aten Primaria 2015;47(8):505–513. [Spanish]	Language not English
Abramson A, Halperin F, Kim, J *et al.* Quantifying the value of orally delivered biologic therapies: a cost-effectiveness analysis of oral semaglutide. J Pharm Sci 2019;108(9):3138-3145	Comparison of dosing/route of administration
Aguiar-Ibáñez R, Palencia R, Kandaswamy P, *et al.* Cost-effectiveness of empagliflozin (Jardiance®) 10 mg and 25 mg administered as an add-on to metformin and sulfonilurea (Met+Su) Compared to other sodium-glucose co-transporter 2 inhibitors (SGLT2Is) in patients with type 2 diabetes mellitus (T2DM). Value Health 2014;17:A351	Abstract only
Aguiar-Ibáñez R, Palencia R, Kandaswamy P, *et al.* Cost-effectiveness of empagliflozin (Jardiance®) 10 mg and 25 mg administered as an add-on to metformin compared to other sodium-glucose cotransporter 2 inhibitors (SGLT2Is) for patients with type 2 diabetes mellitus (T2DM) in the UK. Value Health 2014;17:A350–A351	Duplicate
Ahmad M, Wafai ZA, Khan ZY, *et al.* Evaluation of the cost-effectiveness of different insulin regimes during the peri- operative period in type-2 diabetics in India. J Clin Diagnostic Res 2011;5:1064–1068	Ineligible study design
Alemayehu B, Speiser J, Bloudek L, *et al.* Costs associated with long-acting insulin analogs in patients with diabetes. Am J Manag Care 2018;24(8 Spec No.):SP265-SP272.	Review article
Ashley D, Vega G, Hunt B, *et al.* Evaluating the cost-effectiveness of GLP-1 receptor agonists for the treatment of type 2 diabetes in the UK. Value Health 2015;18(7):A606-A606	Abstract only
Athanasakis K, Zhuo J, Chen J, *et al.* Cost-effectiveness of sitagliptin compared to sulphonylurea as an add-on to metformin in the treatment of type 2 diabetes in Greece. Value Health 2015;18(7):A608-A608	Abstract only
Bacon T, Willis M, Johansen P, *et al.* The cost-effectiveness of canagliflozin verse liraglutide in patients with type 2 diabetes (T2DM) failing to achieve glycaemic control on metformin monotherapy in Ireland. Value Health 2014;17:A345	Abstract only
Bacon T, Willis M, Johansen P, *et al.* The cost-effectiveness of canagliflozin verse insulin-secretagogues (sulphonylureas) or insulin in patients with type 2 diabetes mellitus (T2DM) as an add-on to metformin in Ireland. Value Health 2014;17:A346	Abstract only
Bekele M, Norheim OF, Hailu A. Cost-effectiveness of saxagliptin compared with glibenclamide as a second-line therapy added to metformin for type 2 diabetes mellitus in Ethiopia. MDM Policy Pract 2021;6(1):23814683211005771.	Ineligible outcomes
Brown GC, Brown MM, Turpcu A, Rajput Y. The cost-effectiveness of ranibizumab for the treatment of diabetic macular edema. Ophthalmology 2015;122(7):1416–25	Ineligible study design
CADTH. Glucose replacement agents in frail elderly patients with type ii diabetes in long-term care: clinical and cost-effectiveness, harms, and guidelines. Ottawa: Canadian Agency for Drugs and Technologies in Health (CADTH). Rapid Response. 2015	Review article
Castelo Branco, A, Eriksson M, Nilsson J. Cost-effectiveness analysis of intravitreal aflibercept in diabetic macular oedema in Sweden. Value Health 2015;18(7):A420-A421	Abstract only
Carvalho D, Contente M, Silva C, *et al.* Saxagliptin in the treatment of diabetes mellitus type 2 in Portugal: a study of cost-utility in the perspective of society. Revista Portuguesa da Diabetes 2014;9(2):60–72.	Language not English
Cavusoglu Sezen S, Dokuyucu O, Saylan M, *et al.* Cost-effectiveness of ranibizumab vs. dexamethasone implant in diabetic macular edema. Value Health 2015;18(7):A422-A422	Abstract only
Cazarim MS, da Cruz-Cazarim ELC, Baldoni AO, *et al.* Cost-effectiveness analysis of different dipeptidyl-peptidase 4 inhibitor drugs for treatment of type 2 diabetes mellitus. Diabetes Metab Syndr 2017;11(Suppl 2):S859-S865	Ineligible outcomes
Charokopou M, Vioix H, Eddowes LA, *et al.* Cost-effectiveness of dapagliflozin compared to DPP-4 inhibitors as triple therapy in combination with metformin and a sulphonylurea in the treatment of type 2 diabetes mellitus from a UK health care perspective. Value Health 2014;17:A347	Abstract only
Charokopou M, Vioix H, Verheggen BG, *et al.* Economic assessment of delaying insulin treatment through the use of newer anti-diabetic agents, dapagliflozin (Forxiga(R)) and exenatide (Bydureon(R)), both as add-on to metformin; a cost-effectiveness analysis from a UK NHS perspective. Value Health 2014;17:A344–5	Abstract only
Charokopou M, Vioix H, Verheggen B, *et al.* Cost-effectiveness of dapagliflozin versus DPP-4 inhibitors as monotherapy in the treatment of type 2 diabetes mellitus from a UK health care perspective. Value Health 2014;17: A347	Abstract only
Charokopou M, Vioix H, Verheggen BG, *et al.* Dapagliflozin (Forxiga (R)) versus glipizide as add-on therapies in type 2 diabetes mellitus (T2DM); an update of the cost-effectiveness based on long-term clinical evidence from UK NHS perspective. Value Health 2014;17:A343	Abstract only
Charokopou M, Vioix H, Verheggen B, *et al.* Cost-effectiveness of saxagliptin compared to GLP-1 analogues as an add-on to insulin in the treatment of type 2 diabetes mellitus from a UK health care perspective. Value Health 2014;17(7):A347	Abstract only
Charokopou M, Vioix H, Verheggen BG, *et al.* The impact of long-term clinical evidence on cost-effectiveness of exenatide once weekly (Bydureon®) versus insulin glargine for patients with type 2 diabetes mellitus (T2DM) from a UK NHS perspective. Value Health 2014;17(7):A343	Abstract only
Charokopou M, Chuang L, Verheggen B, *et al.* Cost-effectiveness analysis of exenatide once-weekly versus dulaglutide, liraglutide and lixisenatide for the treatment of type 2 diabetes mellitus: an analysis from the UK NHS Perspective. Value Health 2015;18(7):A606-A606	Abstract only
Chin KL, Ofori-Asenso R, Si S, *et al.* Cost-effectiveness of first-line versus delayed use of combination dapagliflozin and metformin in patients with type 2 diabetes. Sci Rep 2019;9(1):3256	Ineligible outcomes
Chen J, Radican L, Shankar R, *et al.* Cost-effectiveness of sitagliptin versus sulfonylurea as an add-on therapy to metformin in patients with type 2 diabetes in a Belgium setting. Value Health 2014;17(7):A349	Abstract only
Chowdhury EK, Ademi Z, Moss JR, *et al.* Cost-utility of angiotensin-converting enzyme inhibitor-based treatment compared with thiazide diuretic-based treatment for hypertension in elderly Australians considering diabetes as comorbidity. Medicine (Baltimore) 2015;94(9):e590	Ineligible indication
Cleveringa FG, Welsing PM, van den Donk M, *et al.* Cost-effectiveness of the diabetes care protocol, a multifaceted computerized decision support diabetes management intervention that reduces cardiovascular risk. Diabetes Care 2010;33:258–263	Ineligible intervention
Cui J, Klepser DG, McAdam-Marx C. Short-term cost-effectiveness of oral semaglutide for the treatment of type 2 diabetes mellitus in the United States. J Manag Care Spec Pharm 2021;27(7):840-845	Ineligible outcomes
Cui Z, Zhou W, Chang Q, *et al.* Cost-effectiveness of conbercept vs. ranibizumab for age-related macular degeneration, diabetic macular edema, and pathological myopia: population-based cohort study and Markov Model. Front Med (Lausanne) 2021;8:750132	Duplicate
Daacke I, Kandaswamy P, Tebboth A, *et al.* Cost-effectiveness of empagliflozin (jardiance) in the treatment of patients with type 2 diabetes mellitus (T2DM) in the UK based on EMPA-REG outcome data. Value Health 2016;19:A673	Abstract only
Daly MJ, Elvidge J, Chantler T, *et al.* A review of economic models submitted to NICE’s Technology Appraisal Programme, for treatments of T1DM & T2DM. Front Pharmacol 2022;13:887298	Review article
Dawoud D, Fenu E, Wonderling D, *et al.* Basal insulin regimens: systematic review, network meta-analysis, and cost-utility analysis for the National Institute For Health and Care Excellence (NICE) clinical guideline on type 1 diabetes mellitus in adults. Value Health 2015;18(7):A339-A339	Abstract only
Deger C, Ozdemir O, Eldem B, *et al.* The cost-effectiveness (CE) of intravitreal aflibercept (IVT-AFL) in the treatment of diabetic macular edema (DME) in Turkey. Value Health 2015;18(7):A606-A606	Abstract only
Deng J, Gu S, Shao H. Cost-effectiveness analysis of exenatide twice daily (BID) versus insulin glargine once daily (QD) as add-on therapy in Chinese patients with type 2 diabetes mellitus inadequately controlled by oral therapies. Value Health 2015;18(7):A609-A609	Duplicate
DeKoven M, Lee WC, Bouchard, J *et al.* Real-world cost-effectiveness: lower cost of treating patients to glycemic goal with liraglutide versus exenatide. Adv Ther 2014;31(2):202–16	Ineligible outcomes
Dilla T, Alexiou, D, Chatzitheofilou I *et al.* The cost-effectiveness of dulaglutide versus liraglutide for the treatment of type 2 diabetes mellitus in Spain in patients with BMI ≥30 kg/m^2^. J Med Econ 2017;20(5):443-452	Duplicate
Evans M, McEwan P. Clinical and cost-effectiveness of insulin degludec: from clinical trials to clinical practice. J Comp Eff Res 2015;4(3):279-286	Review article
Ektare VU, Lopez JM, Martin SC, *et al.* Cost efficiency of canagliflozin versus sitagliptin for type 2 diabetes mellitus. Am J Manag Care 2014;20(10 Suppl):S204–15	Ineligible outcomes
Gomez AM, Alfonso-Cristancho R, Orozco JJ, *et al.* Clinical and economic benefits of integrated pump/CGM technology therapy in patients with type 1 diabetes in Colombia. Endocrinol Nutr 2016;63(9):466-474	Language not English
Gordon J, McEwan P, Sabale U, *et al.* The cost-effectiveness of exenatide BID versus insulin lispro TID as add-on therapy to titrated insulin glargine in patients with type 2 diabetes - an analysis from the Swedish health care perspective. Value Health 2014;17(7):A344	Abstract only
Granados D, Maurel F, Knudsen M, *et al.* Health economic evaluation of canagliflozin in the treatment of type 2 diabetes mellitus in France. Value Health 2014;17(7):A344	Abstract only
Granström O, Bergenheim K, McEwan P, *et al.* Cost-effectiveness of saxagliptin (Onglyza(R)) in type 2 diabetes in Sweden. Prim Care Diabetes 2012;6(2):127-136.	Duplicate
Gu S, Shen Y, Shi L, *et al.* 1295-P: economic evaluation of drug combination strategy for type 2 diabetes in China. Diabetes Supplement 2019;68	Abstract only
Haig J, Régnier SA, Malcom W, *et al.* Cost-effectiveness of ranibizumab verse aflibercept in treatment of treatment of visual impairment due to diabetic macular oedema (DMO). Value Health 2014;17(7):A609	Abstract only
Halabi A, Nolan M, Marwick T. Cost-effectiveness of strain-guided sodium-glucose co-transporter-2 inhibitors treatment in elderly people with type 2 diabetes mellitus. Heart Lung Circulation 2021;30(Suppl 1):S185-S186	Abstract only
Hayes AJ, Leal J, Gray AM, *et al.* UKPDS Outcomes Model 2: a new version of a model to simulate lifetime health outcomes of patients with type 2 diabetes mellitus using data from the 30 year United Kingdom prospective diabetes study: UKPDS 82. Diabetologia 2013;56(9):1925–1933	A model validation study
Henkhaus LE, Hay JW. Cost effectiveness of empagliflozin/linagliptin as 2nd-line therapy for adults with type 2 diabetes. Value Health 2016;19:A202–A203	Abstract only
Herman WH, Braffett BH, Kuo S, *et al.* The 30-year cost-effectiveness of alternative strategies to achieve excellent glycemic control in type 1 diabetes: An economic simulation informed by the results of the diabetes control and complications trial/epidemiology of diabetes interventions and co. J Diabetes Complications 2018;32(10):934-939	Comparison of dosing/route of administration
Hua YX, Deng Y, Liu M, *et al.* Long-term cost-effectiveness of biphasic human insulin 30 in people with type 2 diabetes with inadequate glycaemic control on oral antidiabetic drugs in China. Value Health 2014;17(7):A745	Abstract only
Hung A, Jois B, Lugo A, *et al.* Cost-effectiveness of diabetes treatment sequences to inform step therapy policies. Am J Manag Care 2020;26(3):e76-e83	Ineligible outcomes
Hunt B, Hansen BB, Ericsson A, *et al.* Evaluation of the cost per patient achieving treatment targets with oral semaglutide: a short term cost-effectiveness analysis in the United States. Adv Ther 2019;36:3483-3493	Ineligible study design
Hutton DW, Stein JD, Bressler NM, *et al.* Cost-effectiveness of intravitreous ranibizumab compared with panretinal photocoagulation for proliferative diabetic retinopathy: secondary analysis from a diabetic retinopathy clinical research network randomized clinical trial. JAMA Ophthalmol. 2017;135(6):576–84	Ineligible study design
Jendle J, Ericsson Å, Gundgaard J, *et al.* Smart insulin pens are associated with improved clinical outcomes at lower cost versus standard-of-care treatment of type 1 diabetes in Sweden: a cost-effectiveness analysis. Diabetes Ther 2021;12(1):373-388	Comparison of dosing/route of administration
Johnston R, Uthman O, Cummins E. Canagliflozin, dapagliflozin and empagliflozin monotherapy for treating type 2 diabetes: systematic review and economic evaluation. Health Technol Assess 2017; 21(2):1-218	Review article
Kansal A, Reifsnider O, Lee J, *et al.* Cost-effectiveness analysis of empagliflozin compared with canagliflozin or standard of care (SoC) in patients with T2DM and established cardiovascular (CV) disease. Diabetes 2018;67	Abstract only
Kim H, Gurrin L, Magliano D, *et al.* Cost utility of diabetes drugs using Hba1c as a direct predictor for quality of life, diabetes complications and mortality. Value Health 2014;17(7):A745-6	Abstract only
Kitano S, Sakamoto T, Goto R, *et al.* The impact of anti-vascular endothelial growth factor agents on visual impairment/blindness prevention in patients with diabetic macular edema and on associated patient and caregiver burden in Japan. J Med Econ 2019;22(3):254-265	Ineligible outcomes
Klimes J, Régnier SA, Mahon R, *et al.* Cost effectiveness analysis of ranibizumab compared to aflibercept and laser intervention in treatment of diabetic macular edema (DME) in the Czech Republic. Value Health 2015;18(7):A419-A419	Abstract only
Kourlaba G, Relakis J, Mahon R, *et al.* Cost-utility of ranibizumab versus aflibercept for treating visual impairment due to diabetic macular edema in Greece. Value Health 2015;18(7):A423-A423	Abstract only
Kousoulakou H, Kalogeropoulou M, Panitti E. Cost-effectiveness analysis of vildagliptin vs. glimepiride as add-on to metformin in the management of type 2 diabetes mellitus in Greece. Value Health 2015;18(7):A608-A608	Abstract only
Krysanov I, Tiapkina M. The long-term cost-effectiveness of twice-daily exenatide with insulin glargine versus once-daily liraglutide with insulin detemir in adult patients with type 2 diabetes in Russia. Value Health 2015;18(7):A606-A606	Abstract only
Kymes SM. Incremental cost-effectiveness of proliferative diabetic retinopathy treatments: the certainty of uncertainty. JAMA Ophthalmol 2017;135(6):584-585	Editorial/commentary/summary
Kwon CS, Seoane-Vazquez E, Rodriguez-Monguio R. Cost-effectiveness analysis of metformin+dipeptidyl peptidase-4 inhibitors compared to metformin+sulfonylureas for treatment of type 2 diabetes. BMC Health Serv Res 2018;18(1):78	Ineligible outcomes
Lambadiari V, Ozdemir Saltik AZ, de Portu S, *et al.* Cost-effectiveness analysis of an advanced hybrid closed-loop insulin delivery system in people with type 1 diabetes in Greece. Diabetes Technol Ther 2022;24(5):316-323	Comparison of dosing/route of administration
Lamotte M, Salem A, Mettam SR, *et al.* 1292-P: Projected long-term clinical benefit and cost-effectiveness of empagliflozin compared with glimepiride in patients with type 2 diabetes in China. Diabetes 2019;68	Abstract only
Langer J, Hunt B, Valentine WJ. Evaluating the short-term cost-effectiveness of liraglutide versus sitagliptin in patients with type 2 diabetes failing metformin monotherapy in the United States. J Manag Care Pharm 2013;19(3):237–46	Ineligible outcomes
Li R, Zhang P, Barker LE, Hoerger TJ. Cost-effectiveness of aspirin use among persons with newly diagnosed type 2 diabetes. Diabetes Care 2010;33:1193–1199	Ineligible intervention
Li, Q, Chitnis, A, Hammer, M, *et al.* Real-world clinical and economic outcomes of liraglutide versus sitagliptin in patients with type 2 diabetes mellitus in the United States. Diabetes Ther 2014;5(2):579–590	Ineligible study design
Lin J, Chang JS, Smiddy WE. Cost evaluation of panretinal photocoagulation versus intravitreal ranibizumab for proliferative diabetic retinopathy. Ophthalmology 2016;123(9):1912–8.	Ineligible outcomes
Lundqvist A, Carlsson KS, Johansen P, Andersson E, Willis M. A model validation study of the IHE cohort model of type 2 diabetes and the impact of choice of macrovascular risk equation. PLoS One. 2014;9(10):e110235	A model validation study
Ly TT, Brnabic AJ, Eggleston A, *et al.* A cost-effectiveness analysis of sensor-augmented insulin pump therapy and automated insulin suspension versus standard pump therapy for hypoglycemic unaware patients with type 1 diabetes. Value Health 2014;17(5):561-9	Comparison of dosing/route of administration
Mettam SR, Bajaj H, Kansal AR, *et al.* Cost effectiveness of empagliflozin in patients with T2DM and high CV risk in Canada. Value Health 2016;19:A674	Abstract only
McQueen RB, Ellis SL, Campbell JD, *et al.* Cost-effectiveness of continuous glucose monitoring and intensive insulin therapy for type 1 diabetes. Cost Eff Resour Alloc 2011;9:13	Comparison of dosing/route of administration
Navarro-Navarro A, Salom D, Martínez-Toldos JJ, *et al.* The diabetic retinopathy clinical research network analysis of the cost-effectiveness of aflibercept, bevacizumab and ranibizumab for the treatment of diabetic macular oedema and its application in Spain. Arch Soc Esp Oftalmol 2017;92(5):245-246	Editorial/commentary/summary
Nguyen HV, Schatz DA, Mital S, *et al.* Cost-effectiveness of low-dose antithymocyte globulin versus other immunotherapies for treatment of new-onset type 1 diabetes. Diabetes Technol Ther 2022;24(4):258-267	Ineligible intervention
National Institute for Health and Care Excellence. Canagliflozin, dapagliflozin and empagliflozin as monotherapies for treating type 2 diabetes. Technology appraisal guidance [TA390]. 25 May 2016. https://www.nice.org.uk/guidance/ta390	Review article
National Institute for Health and Care Excellence. Ertugliflozin as monotherapy or with metformin for treating type 2 diabetes. Technology appraisal guidance [TA572]. 27 March 2019. https://www.nice.org.uk/guidance/ta572	Ineligible study design
National Institute for Health and Care Excellence. Ertugliflozin with metformin and a dipeptidyl peptidase-4 inhibitor for treating type 2 diabetes. Technology appraisal guidance [TA583]. 05 June 2019. https://www.nice.org.uk/guidance/ta583.	Ineligible study design
National Institute for Health and Care Excellence. Fluocinolone acetonide intravitreal implant for treating chronic diabetic macular oedema in phakic eyes after an inadequate response to previous therapy. Technology appraisal guidance [TA613]. 20 November 2019. https://www.nice.org.uk/guidance/ta613	Health technology assessment report without (sufficient) modeling description
National Institute for Health and Care Excellence. Faricimab for treating diabetic macular oedema. Technology appraisal guidance [TA799]. 29 June 2022. https://www.nice.org.uk/guidance/ta799.	Ineligible study design
National Institute for Health and Care Excellence (NICE) 2021 Jul. Ref ID: 34464036	Review article
Oksuz E, Malhan S, Kamaci E, *et al.* Cost-effectiveness of empagliflozin (Jardiance®) in the treatment of patients with type 2 diabetes mellitus (T2DM) in Turkey based EMPA-REG outcome data. Value Health 2017;20:A479	Abstract only
Pawaskar M, Bilir SP, Davies GM. 1275-P: Cost effectiveness of using DPP-4i and SGLT2i combination therapy vs. switching to GLP-1 therapy for the management of type 2 diabetes. Diabetes Supplement 2019;68.	Duplicate
Pawlik D, Wójcik R, Zawodnik A, *et al.* Cost-effectiveness of empagliflozin in patients with type 2 diabetes mellitus at high cardiovascular risk in Poland. Value Health 2017;20:A478	Abstract only
Pereira, R, Gouveia, M, Martins, AP. Ana´lise custo-efectividade de sitagliptina quando adicionada a metformina em doentescom diabetes tipo 2 em Portugal. Revista Portuguesa de diabetes 2012;7(1): 13–23	Language not English
Permsuwan U, Dilokthornsakul P, Thavorn K, *et al.* Cost-effectiveness of dipeptidyl peptidase-4 inhibitor monotherapy versus sulfonylurea monotherapy for people with type 2 diabetes and chronic kidney disease in Thailand. J Med Econ 2017;20(2):171-181.	Ineligible indication
Pititto L, Neslusan C, Teschemaker AR, *et al.* Cost-effectiveness of canagliflozin (CANA) versus sitagliptin (SITA) as add-on to metformin plus sulfonylurea in patients with type 2 diabetes mellitus (T2DM) in Brazil. Value Health 2015;18:A864	Abstract only
Pollock RF, Tikkanen CK. A short-term cost-utility analysis of insulin degludec versus insulin glargine U100 in patients with type 1 or type 2 diabetes in Denmark. J Med Econ 2017;20(3):213-220.	Duplicate
Raibouaa A, Borgeke H, Alexiou D, *et al.* Cost-effectiveness of dulaglutide 1.5mg once weekly for the treatment of patients with type two diabetes mellitus in Sweden. Value Health 2015;18(7):A607-A607	Abstract only
Ramírez de Arellano A, Lizán L, Prades M, *et al.* Cost-effectiveness analysis of insulin detemir versus insulin neutral protamine Hagedorn (NPH) in patients with type 1 diabetes mellitus in Spain. Value Health 2014;17(7):A343	Abstract only
Ramírez de Arellano A, Morales C, De LD, *et al.* Short-term cost-effectiveness analysis of insulin detemir versus insulin neutral protamine Hagedorn (NPH) in patients with type 2 diabetes mellitus in Spain. Value Health 2014;17(7):A343	Abstract only
Ramsey DJ, Poulin SJ, LaMonica LC, *et al.* Early conversion to aflibercept for persistent diabetic macular edema results in better visual outcomes and lower treatment costs. Clin Ophthalmol 2021;15:31-39	Comparison of dosing/route of administration
Reifsnider O, Pimple P, Stargardter MJD, *et al.* 1158-P: Cost-effectiveness of empagliflozin vs. liraglutide as second-line therapy for type 2 diabetes in the United States. Diabetes Supplement 2020;69	Abstract only
Riemsma R, Corro Ramos I, Birnie R, *et al.* Integrated sensor-augmented pump therapy systems [the MiniMed® Paradigm™ Veo system and the Vibe™ and G4® PLATINUM CGM (continuous glucose monitoring) system] for managing blood glucose levels in type 1 diabetes: a systematic review and economic evaluation. Health Technol Assess 2016;20(17):v-xxxi, 1-251	Comparison of dosing/route of administration
Rosselli D, Quitian H, Gomez AM, *et al.* Cost-utility analysis of insulin analogues compared with multiple daily injections of human insulin for the treatment of 15 years old or older patients with type 1 diabetes mellitus in Colombia. Value Health 2015;18(7):A609-A609	Abstract only
Roze S, Duteil E, Smith-Palmer J, *et al.* Cost-effectiveness of continuous subcutaneous insulin infusion in people with type 2 diabetes in the Netherlands. J Med Econ 2016;19(8):742–749	Comparison of dosing/route of administration
Roze S, Smith-Palmer J, Valentine WJ, *et al.* Long-term health economic benefits of sensor-augmented pump therapy vs continuous subcutaneous insulin infusion alone in type 1 diabetes: a U.K. perspective. J Med Econ 2016;19(3):236-42	Comparison of dosing/route of administration
Roze S, Smith-Palmer J, Delbaere A, *et al.* Cost-effectiveness of continuous subcutaneous insulin infusion versus multiple daily injections in patients with poorly controlled type 2 diabetes in Finland. Diabetes Ther 2019;10(2):563-574	Comparison of dosing/route of administration
Ruiz MC, Ubiarco LV. Cost-effectiveness of ranibizumab on patients with diffuse diabetic macular edema within the public Mexican health care system. Value Health 2014;17(7):A607	Abstract only
Sánchez-Covisa J, Capel M, Baeten S, *et al.* Comparative cost-effectiveness analysis of adding twice-daily exenatide to insulin glargine versus adding insulin lispro to treat type 2 diabetes in Spain. Value Health 2014;17(7):A349-50	Abstract only
Sánchez-Covisa J, Capel M, Schmidt R, *et al.* The cost-effectiveness of dapagliflozin in combination with insulin for the treatment of type 2 diabetes mellitus (T2DM) in Spain. Value Health 2014;17:A350	Abstract only
Sánchez-Covisa J, Franch J, Mauricio D, *et al.* The cost-effectiveness of saxagliptin when added to metformin and sulphonylurea in the treatment of type 2 diabetes mellitus In Spain. Value Health 2014;17(7):A350.	Abstract only
Sánchez-Covisa J, Franch J, Mauricio D, *et al.* Análisis de coste-efectividad de saxagliptina como tratamiento triple oral (con metformina y una sulfonilurea) en el manejo de la diabetes tipo 2 en España. Pharmacoeconomics 2015;13(1):25–35	Language not English
Sánchez-Covisa J, Franch J, Mauricio D, *et al.* Cost-effectiveness analysis of saxagliptin as oral triple therapy (with metformin and a sulfonylurea) in the management of type 2 diabetes in Spain. Pharmacoeconomics - Spanish Research Articles 2016;13(1):25-35	Language not English
Saunders R, Boye KS, van Brunt K, *et al.* Cost-effectiveness of rapid-acting analog insulin for type 1 diabetes in the UK setting. Value Health 2015;18(7):A610-A610	Abstract only
Schroeder M, Johansen P, Thompson G, *et al.* The cost-effectiveness of canagliflozin (cana) versus dapagliflozin (DAPA) in patients with type 2 diabetes mellitus (T2DM) with inadequate control on metformin (Met) monotherapy in the United Kingdom. Value Health 2014;17(7):A344	Abstract only
Schroeder M, Johansen P, Willis M, *et al.* The cost-effectiveness of canagliflozin (CANA) versus dapagliflozin (DAPA) 10mg and empagliflozin (EMPA) 25mg in patients with type 2 diabetes mellitus (T2DM) as monotherapy in the United Kingdom. Value Health 2015;18:A607	Abstract only
Shi LW, Han S, Liu F. Evaluating the long-term cost-effectiveness of liraglutide 1.2 mg and exenatide in patients with type 2 diabetes mellitus. Value Health 2014;17(7):A744-5	Abstract only
Shyangdan D, Cummins E, Royle P, *et al.* Liraglutide for the treatment of type 2 diabetes. Health Technol Assess 2011;15(Suppl 1):77-86	Health technology assessment report without (sufficient) modeling description
Shyangdan D, Cummins E, Royle P, *et al.* Liraglutide for the treatment of type 2 diabetes. Health Technol Assess May 2011;15(Suppl 1):77-86	Duplicate
Sivaprasad S, Prevost AT, Bainbridge J, *et al.* Clinical efficacy and mechanistic evaluation of aflibercept for proliferative diabetic retinopathy (acronym CLARITY): a multicentre phase IIb randomised active-controlled clinical trial. BMJ Open 2015;5(9):e008405	Protocol
Su ZT, Bartelt-Hofer J, Brown S, *et al.* The use of computer simulation modeling to estimate complications in patients with type 2 diabetes mellitus: comparative validation of the Cornerstone Diabetes Simulation model. Pharmacoecon Open 2020;4(1):37-44	A model validation study
Szmurlo D, Drzal R, Plisko R, *et al.* Cost effectiveness evaluation of canagliflozin in combination with metformin and sulfonylurea in comparison to NPH insulin in the treatment of type 2 diabetes mellitus in Poland. Value Health 2014;17:A351	Abstract only
Szmurlo D, Drzal R, Plisko R, *et al.* Cost effectiveness evaluation of canagliflozin in combination with metformin in the treatment of type 2 diabetes mellitus in Poland. Value Health 2014;17:A346	Abstract only
Tamilselvan T, Kumutha T, Lekshmi VA, *et al.* Pharmacoeconomical evaluation of oral hypoglycemic agents for type-2 diabetes mellitus in a multispeciality hospital. Int J Pharm Sci Res 2017;8:2243-2248	Ineligible outcomes
Tandon T, Dubey AK, Srivastava S, *et al.* A pharmaoeconomic analysis to compare cost-effectiveness of metformin plus teneligliptin with metformin plus glimepiride in patients of type-2 diabetes mellitus. J Family Med Prim Care 2019;8:955-959.	Ineligible outcomes
Teramachi H, Ohta H, Tachi T, *et al.* Pharmacoeconomic analysis of DPP-4 inhibitors. Pharmazie 2013;68(11):909–15	Ineligible outcomes
Thayer S, Wei W, Buysman E, *et al.* The INITIATOR study: pilot data on real-world clinical and economic outcomes in US patients with type 2 diabetes initiating injectable therapy. Adv Ther 2013;30(12):1128–40	Ineligible study design
Tikkanen CK, Johansen P, Hunt B, *et al.* Once-weekly semaglutide provides better health outcomes compared to dulaglutide as dual therapy in the treatment of type 2 diabetes: a cost-effectiveness analysis. Management 2018;12(776):12-640	A poster
Troelsgaard A, Huetson P, Kjellberg J, *et al.* Health economic evaluation of canagliflozin in the treatment of type 2 diabetes mellitus in Norway. Value Health 2014;17(7):A345	Abstract only
Troelsgaard A, Knudsen M, Maia-Lopes S, *et al.* health economic evaluation of canagliflozin in the treatment of type 2 diabetes mellitus in Portugal. Value Health 2014;17(7):A343-4.	Abstract only
Troelsgaard A, Pitcher A, Binder R, *et al.* Health economic evaluation of canagliflozin in the treatment of type 2 diabetes mellitus in Slovakia. Value Health 2014;17(7):A345	Abstract only
Troelsgaard A, Pitcher A, Granados D, *et al.* The cost-effectiveness of canagliflozin compared with liraglutide in patients with type 2 diabetes inadequately controlled with metformin and sulfonylurea in France. Value Health 2014;17:A346–7	Abstract only
Troelsgaard A, Pitcher A, Veselá Š, *et al.* Health economic evaluation of canagliflozin in the treatment of type 2 diabetes mellitus in Czech Republic. Value Health 2014;17(7):A342-3	Abstract only
Tzanetakos C, Tentolouris N, Kourlaba G, *et al.* Cost-effectiveness of dapagliflozin as add-on to metformin for the treatment of type 2 diabetes in Greece. Value Health 2015;18(7):A606-A607	Abstract only
Tzanetakos C, Melidonis A, Verras C. Cost-effectiveness analysis of liraglutide versus sitagliptin or exenatide in patients with inadequately controlled type 2 diabetes on oral antidiabetic drugs in Greece. Value Health 2014;17(7):A345	Duplicate
Valentine WJ, Curtis BH, Pollock RF, *et al.* Is the current standard of care leading to cost-effective outcomes for patients with type 2 diabetes requiring insulin? A long-term health economic analysis for the UK. Diabetes Res Clin Pract 2015;109(1):95-103	Comparison of dosing/route of administration
Wang B, Roth JA, Nguyen H, *et al.* The short-term cost-effectiveness of once-daily liraglutide versus once-weekly exenatide for the treatment of type 2 diabetes mellitus in the United States. PLoS One 2015;10(4):e0121915	Ineligible outcomes
Willis M, Asseburg C, He J. A model validation study of economic and health outcomes simulation model of type 2 diabetes mellitus (ECHO-T2DM). J Med Econ 2013;16(8):1007–1021	A model validation study
Woo V, Zinman B, Pieber TR. 179 - short-term cost-effectiveness of insulin degludec vs. insulin glargine 100 units/ml for patients with type 2 diabetes at high risk of hypoglycemia in DEVOTE…Diabetes Canada/Canadian Society of Endocrinology and Metabolism Professional Conference. Can J Diabetes 2018;42:S60-S60	Abstract only
Wu B, Ma J, Zhang S, Zhou L, Wu H. Development and validation of a health policy model of type 2 diabetes in Chinese setting. J Comp Eff Res 2018;7(8):749-763	A model validation study
Zupa M, Codario R, Smith KJ. 815-P: Cost-effectiveness of subcutaneous semaglutide vs. empagliflozin as add-on therapy for type 2 diabetes. Diabetes Supplement 2021;70	Abstract only

## Appendix III: Data extraction instrument

**Table:**

**Scoping review details**
Scoping review title:
Review objective/s:
Review question/s:
**Evidence source details and characteristics** (in relation to the population and sources of the scoping review)
Citation details (eg, author/s, date, title, journal, volume, issue, pages)
Country (free text)
Population/condition (T1DM, T2DM, DR, DME)
**Types of sources**
Comparators (free text)
Modeling method
Model (Cardiff T1DM, Cardiff T2DM, IQVIA CORE Diabetes Model, UKPDS-OM2, IHECM T2DM, Chinese Outcomes Model for T2DM (COMT), ECHO-T2DM, the PRIME model, CRC DES model, model by NICE, model by Kansal *et al.*, model by Viriato *et al.*, model by Ericsson *et al.*, model by Ridderstråle *et al.*, model by Valentine *et al.*, model by Abushanab *et al.*, model self-made by authors, NICE/CADTH HTA)
Time horizon of the analysis (years)
Perspective of the analysis (societal/health care (irrespective of the funder))
Discounting applied in the analysis (yes/no, rate used %)
Source of efficacy results
PROM used for measuring quality of life or effectiveness (PROM instrument)
Result of the CEA or CUA (ICER and qualitative conclusion of the result)
**Details/results extracted from source of evidence (in relation to the concept of the scoping review)**
Concept
ADEs incorporated to the analysis? (yes/partly/no)
**If no**: Are ADEs reported in the study that provides the efficacy estimates for the CEA/CUA*? (yes/no), Reasons for not including ADEs in the analysis [free text]
**If yes:**
Which adverse drug events [free text]
The source of the incidence of ADEs [free text, eg, RCT, meta-analysis]
How included (expected values, submodel, separate health state, risk equation)
Adverse drug events in cost estimates (yes/partly/no), source of costs [free text]
Adverse drug events in quality-of-life estimates (yes/no, source of the estimate and PROM used)
Additional details of incorporation of ADEs [free text, eg, thresholds for severity/incidence for included ADEs]
**If partly**: [free text, explain why “partly”]
Discussion of the adverse drug events and results (does the study discuss the role of ADEs in the analysis Are ADEs addressed in the sensitivity analyses, if so, what their impact seems to be in relation to the numeric and descriptive results of the analysis)

ADE, adverse drug events; CEA, cost-effectiveness analyses; CUA, cost-utility analysis; DME, diabetic macular edema; DR, diabetic retinopathy; ICER, incremental cost-effectiveness ratio; PROM, patient-reported outcome measure; RCT, randomized controlled trial; T1DM, type 1 diabetes mellitus; T2DM, type 2 diabetes mellitus.

## Appendix IV: Characteristics of included studies

**Table:**

Study/country	Population/condition	Comparators	Modeling method	Model^a^	Time horizon	Perspective	Discount rate	Source of efficacy results	PROM used	ICER^b^	Results [free text]
Abushanab *et al.* 2022a^31^ Qatar	T2DM	Empagliflozin + metformin vs SOC	Markov model	17	Lifetime	Health care payer	3%	Clinical trial (EMPA REG OUTCOME)	EQ-5D	QAR 39,245/QALY	Empagliflozin combined with metformin cost-effective
Abushanab *et al.* 2022b^32^ Australia	T2DM	Empagliflozin + metformin vs SOC	Markov model	17	5 years	Health care payer + societal	5%	Clinical trial (EMPA REG OUTCOME)	EQ-5D	A$28,244/QALY	Empagliflozin + metformin may be cost-effective
Bagepally *et al.* 2021^33^ India	T2DM	Dapagliflozin vs Sulfonylureas	Markov model	18	Lifetime	Health care payer	3%	Clinical trial (Nauck *et al.* 2014)	Unknown	$1421/QALY	Dapagliflozin cost-effective compared to sulfonylureas
Bain *et al.* 2020^34^ UK	T2DM	Semaglutide vs empagliflozin, sitagliptin, liraglutide	Simulation model	3	Lifetime (50 years)	Health care payer	0% and 6%	Clinical trial (PIONEER 2, 3 & 4)	EQ-5D (Clarke *et al.*)	Empa: £11,006 Sita: £4930	Oral semaglutide 14 mg cost-effective vs empagliflozin 25 mg, sitagliptin 100 mg, and liraglutide 1.8 mg
Barnett *et al.* 2018^35^ UK	T2DM	Liraglutide vs sitagliptin	Simulation model	3	Lifetime (50 years)	Health care payer	3.5%	Clinical trial (LIRA-SWITCH)	Unknown	£15,423/QALY	Switching from sitagliptin 100 mg to liraglutide 1.8 mg is likely to be considered cost-effective
Basson *et al.* 2018^36^ France	T2DM	Dulaglutide vs exenatide	Simulation model	3	Lifetime (40 years)	Health care payer	4%	NMA, clinical trial (AWARD)	EQ-5D (Beaudet *et al.* 2014)	Dominant	Dulaglutide 1.5 mg dominant compared to exenatide QW
Beaudet *et al.* 2011^37^ UK	T2DM	Exenatide vs insulin glargine	Semi-Markov	3	Lifetime (50 years)	Health care payer	3.5%	Clinical trial (DURATION-3)	EQ-5D (Clarke *et al.*)	£10,597/QALY	At a price equivalent to liraglutide 1.2 mg, exenatide QW was more effective and more costly than insulin glargine
Becker *et al.* 2022^38^ US, UK	T2DM	Exenatide vs placebo	Simulation model	4	Lifetime	Health care payer	US: 3% UK: 3.5%	Clinical trial (EXSCEL)	EQ-5D	US: $259,223/QALY UK: £42,589/QALY	Adding exenatide QW to usual care increased QALYs and costs compared with usual care alone
Bennett *et al.* 2020^39^ UK	T1DM	Dapagliflozin + insulin vs insulin	Simulation model	1	Lifetime	Health care payer	3.5%	Clinical trial (DEPICT-1+2), NMA	Peasgood *et al.* (EQ-5D)	£10,143/QALY	Dapagliflozin as an adjunct to insulin is cost-effective
Bergenheim *et al.* 2012^40^ US	T2DM	Saxagliptin vs sulfonylurea (glipizide)	Simulation model	2	Lifetime	Health care payer	3%	Clinical trial (Göke *et al.*)	Unknown	Dominant	Saxagliptin is a cost-effective treatment option
Brown *et al.* 2014^41^ Canada	T2DM	Insulin glargine vs sitagliptin	Semi-Markov	3	Lifetime (50 years)	Health care payer	5%	Clinical trial (EASIE)	EQ-5D (Clarke *et al.*)	Dominant	Insulin glargine is a cost-effective alternative to sitagliptin
Bruhn *et al.* 2016^42^ US	T2DM	Albiglutide vs insulin lispro, insulin glargine, sitagliptin	Semi-Markov	3	50 years	Health care payer	0% and 6%	Clinical trial (HARMONY 6, 4 and 3)	EQ-5D (Clarke *et al.*)	$43,541/QALY $79,166/QALY $22,094/QALY	Albiglutide is a reasonable treatment option based on cost-utility, relative to insulin lispro, insulin glargine, and sitagliptin
Brändle *et al.* 2011^43^ Switzerland	T2DM	Insulin glargine vs NPH insulin	Simulation model	9	40 years	Health care payer	3.5%	Meta-regression	EQ-5D (Clarke *et al.*)	CHF 26,271/QALY	Insulin glargine proved to be cost-effective
CADTH 2013^44^ Canada	T2DM	Sulfonylureas, meglitinides, TZDs, GLP-1 analogs, DPP-4 inhibitors, insulins, alpha-glucosidase inhibitors	Simulation model	19	40 years	Health care payer	Not reported	NMA	EQ-5D	Several ICERs	The addition of a sulfonylurea to metformin had the highest probability of being the most cost-effective strategy
Cai *et al.* 2019^45^ China	T2DM	Dapagliflozin vs metformin	Simulation model	2	Lifetime	Health care payer	3%	Meta-analysis and indirect comparison	EQ-5D (Clarke *et al.*)	CNY 10,729/QALY	Dapagliflozin cost-effective compared with metformin
Capehorn *et al.* 2021^46^ UK	T2DM	Semaglutide vs empagliflozin	Simulation model	3	Lifetime	Health care payer	3.5%	Meta-analysis	EQ-5D (Clarke *et al.*)	£4439/QALY	QW semaglutide 1 mg cost-effective compared with empagliflozin 25 mg
Capel *et al.* 2020^47^ Spain	T2DM	Exenatide vs GLP-1 RA	Simulation model	2	40 years	Health care payer	3%	Indirect comparison	EQ-5D	Dominant	Exenatide 2 mg/week dominant vs the other GLP-1 RAs
Cardoso *et al.* 2016^48^ Portugal	T1DM and T2DM	Insulin detemir vs NPH	No specification	15, 16	1 year	Health care payer	No discounting	A systematic review and meta-analysis	Unknown	T1DM: €2096 to 9936/QALY T2DM: €4145 to 19,999/QALY	Insulin detemir cost-effective alternative to NPH
Carvalho *et al.* 2021^49^ Portugal	T2DM	Semaglutide vs empagliflozin	Simulation model	3	Lifetime	Health care payer	4%	Meta-analysis	EQ-5D	€14,114/QALY	Semaglutide was cost-effective
Chakravarty *et al.* 2018^50^ US	T2DM	Dapagliflozin vs GLP-1 RA, sulfonylurea, DPP-4-inhibitors, TZD	No specification	18	1 year	Health care payer	No discounting	NMA	Unknown	Many ICERs	Dapagliflozin was cost-saving compared with GLP-1 RAs and DPP-4 inhibitors, and cost-effective compared with sulfonylurea and TZD
Chalk *et al.* 2014^51^ UK	DME	Bevacizumab vs laser therapy	Markov model	18	4 years	Health care payer	3.5%	Clinical trial (The BOLT study)	EQ-5D	£51,182/QALY	Bevacizumab is not cost-effective
Charokopou *et al.* 2015a^52^ UK	T2DM	Dapagliflozin vs sulfonylureas	Simulation model	2	Lifetime (40 years)	Health care payer	3.5%	Clinical trial (Nauck *et al.* 2014)	EQ-5D	£2671/QALY	Dapagliflozin in combination with metformin is cost-effective compared with sulfonylureas
Charokopou *et al.* 2015b^53^ UK	T2DM	Dapagliflozin vs DPP-4 inhibitors	Simulation model	2	Lifetime	Health care payer	3.5%	NMA (Goring)	EQ-5D	£6761/QALY	Dapagliflozin in combination with metformin is cost-effective
Cheng *et al.* 2019^54^ China	T2DM	Insulin degludec vs insulin glargine	Simulation model	6	Lifetime	Health care payer	5%	Clinical trial	EQ-5D	$613,443/QALY	Insulin degludec unlikely to be cost-effective compared with insulin glargine
Chien *et al.* 2020^55^ Taiwan	T2DM	SGLT2 inhibitors, GLP-1 RA, DPP-4 inhibitors, sulfonylurea, and insulin	Simulation model	2	40 years	Health care payer	3%	NMA, RCT	Unknown	Several ICERs	The addition of either sulfonylurea or SGLT2 inhibitors to metformin was found to be cost-effective
Choi *et al.* 2022^56^ US	T2DM	SGLT2 inhibitors/GLP-1 RAs vs metformin	Microsimulation	4	Lifetime	Health care payer	3%	Unknown	EQ-5D (Clarke *et al.*)	SGLT2 inhibitors: 478,000/QALY GLP-1 RAs: inferior	SGLT2 inhibitors and GLP-1 RAs would improve T2DM outcomes, but their costs would need to fall by at least 70% to be cost-effective
Chuang *et al.* 2016^57^ UK	T2DM	Exenatide vs dulaglutide, liraglutide, lixisenatide	Simulation model	2	Lifetime (40 years)	Health care payer	3%	NMA	EQ-5D	Several ICERs	Exenatide is cost-effective compared to dulaglutide, liraglutide, and lixisenatide
Cui *et al.* 2021^58^ China	DME	Conbercept vs ranibizumab	Markov model	18	10 years	Health care payer	3.5%	Clinical trial	TTO	RMB −258,813/QALY	Conbercept is suitable and cost-effective in treatment of DME compared with ranibizumab
Cutino *et al.* 2015^59^ US	DME	Fluocinolone acetonide vs sham	Markov model	18	15 years	Societal	Unknown	Clinical trial (FAME-study)	TTO	<$50,000/QALY	Fluocinolone acetonide implant is cost-effective
Davies *et al.* 2012^60^ UK	T2DM	Liraglutide vs glimepiride or sitagliptin	Semi-Markov	3	Lifetime	Health care payer	3.5%	Clinical trial (LEAD-2)	EQ-5D	Several ICERs	Liraglutide, added to metformin monotherapy, is cost-effective
Davies *et al.* 2016^61^ UK	T2DM	IDegLira vs alternative basal insulin	Semi-Markov	3	Lifetime (40 years)	Health care payer	3.5%	Clinical trial (DUAL-V)	EQ-5D	£6090/QALY	IDegLira may be considered cost-effective
Dawoud *et al.* 2017^62^ UK	T1DM	Basal insulin regimens	Semi-Markov	3	Lifetime	Health care payer	3.5%	SLR, NMA	EQ-5D (Clarke *et al.*)	Several ICERs	Insulin detemir (twice daily) is the most cost-effective regimen
Deerochanawong *et al.* 2021^63^ Thailand	T2DM	Dapagliflozin + SOC vs SOC	Semi-Markov	18	Lifetime	Societal	3%	Clinical trial (DECLARE-TIMI 58)	EQ-5D (Clarke *et al.*)	$18,988	Dapagliflozin results in an ICER which exceeds the local threshold of 5310 USD/QALY
Dempsey *et al.* 2018a^64^ US	T2DM	IDegLira vs Insulin glargine	Simulation model	3	Lifetime	Health care payer	3%	Clinical trial (DUAL-IIV[open-label])	EQ-5D (Clarke *et al.*)	Dominant	IDegLira is likely to be considered dominant
Dempsey *et al.* 2018b^65^ US	T2DM	IDegLira vs insulin glargine + insulin aspart	No specification	18	1 year	Health care payer	No discounting	Clinical trial (DUAL-VII)	Unknown	Dominant	IDegLira is dominant
Deng *et al.* 2015^66^ China	T2DM	Exenatide vs insulin glargine	Simulation model	2	40 years	Societal	3%	Meta-analysis	EQ-5D (Clarke *et al.*)	RMB 61,078/QALY	Compared with insulin glargine once daily, exenatide twice daily as add-on therapy to oral antidiabetic agent is a cost-effective treatment
Dewan *et al.* 2012^67^ N/A	DME	Ranibizumab + laser vs triamcinolone + laser	Microsimulation	18	2 years	Health care payer	Unknown	Clinical trial	Unknown (converted from visual acuity)	Several ICERs	Triamcinolone seems to be the most cost-effective option
Dilla *et al.* 2017^68^ Spain	T2DM	Dulaglutide vs liraglutide	Semi-Markov	3	Lifetime	Health care payer	3%	Clinical trial (AWARD-6)	EQ-5D (Beaudet *et al.* 2014)	Dominant	Dulaglutide 1.5 mg was more effective and less costly than liraglutide 1.8 mg
Drummond *et al.* 2018^69^ UK	T2DM	IDegLira vs insulin glargine + insulin aspart	No specification	18	1 year	Health care payer	No discounting	Clinical trial (DUAL-IIV[open-label])	Unknown	£5924/QALY	IDegLira is a cost-effective alternative to basal-bolus therapy with insulin glargine U100 plus insulin aspart
Ehlers *et al.* 2021^70^ Denmark	T2DM	Empagliflozin vs liraglutide	Simulation model	3	Lifetime (50 years) and 5 years	Health care payer	4%	Clinical trial (EMPA-REG OUTCOME)	EQ-5D (Beaudet *et al.* 2014)	Dominant	The cost-effectiveness analysis suggests that empagliflozin plus SOC is dominant compared to liraglutide plus SOC
Ehlers *et al.* 2022a^71^ Denmark	T2DM	Oral semaglutide vs empagliflozin	Simulation model	3	Lifetime (50 years)	Health care payer	4%	Clinical trial (PIONEER 2)	EQ-5D (Beaudet *et al.* 2014)	DKK 1,930,548/QALY	Oral semaglutide + metformin seems not cost-effective vs empagliflozin + metformin
Ehlers *et al.* 2022b^72^ Denmark	T2DM	Subcutaneous semaglutide vs empagliflozin	Simulation model	3	50 years	Health care payer	4%	Clinical trial (SUSTAIN 2, 3 and 8, PIONEER 2)	EQ-5D (Beaudet *et al.* 2014)	€100,239/QALY	Subcutaneous semaglutide plus metformin is not cost-effective compared to empagliflozin plus metformin
Ekhlasi *et al.* 2022^73^ Iran	T2DM	Dulaglutide vs liraglutide	Markov model	18	10 years	Health care payer	7.4% (costs) and 5% (costs)	Meta-analysis (Taheri *et al.*)	Standard gamble (Boye *et al.*)	Dominant	Dulaglutide is cost-effective
Elgart *et al.* 2013^74^ Argentina	T2DM	Saxagliptin vs sulfonylurea	Discrete event simulation model	2	20 years	Health care payer	3.5%	Head-to-head trial (Göke *et al.*)	EQ-5D	$20,490 /QALY	The combination saxagliptin + metformin is highly cost-effective
Eliasson *et al.* 2022^75^ Sweden	T2DM	Oral semaglutide vs empagliflozin and sitagliptin	Markov model	5	40 years	Health care payer and societal	3%	Clinical trial (PIONEER 2 and 3)	EQ-5D (Beaudet *et al.* 2014)	Empa: SEK 191,721/QALY Sita: SEK 95,234/QALY	Oral semaglutide was cost-effective compared with empagliflozin and sitagliptin
Erhardt *et al.* 2012^76^ Germany	T2DM	Saxagliptin vs sulfonylurea	Discrete event simulation model	2	40 years	Societal	3%	SLR, meta-analysis, head-to-head trial	EQ-5D	€13,931/QALY	Improved outcomes with saxagliptin at a cost that would likely be considered acceptable
Ericsson *et al.* 2013^77^ Sweden	T1DM and T2DM	Insulin degludec vs insulin glargine	Markov model	14	1 year	Societal	No discounting	Meta-analysis, self-reported questionnaire	TTO	Many ICERs	Degludec is likely to be cost-effective compared to glargine
Ericsson *et al.* 2018^78^ Sweden	T2DM	Liraglutide vs lixisenatide	Markov model	5	40 years	Societal	3%	ITC	TTO	Dominant	Liraglutide and IDegLira were cost-saving
Ericsson & Fridhammar 2019^79^ Sweden	T2DM	Semaglutide vs dulaglutide and lixisenatide	Markov model	5	40 years	Societal	3%	Clinical trial (SUSTAIN 7), NMA	EQ-5D (Kiadaliri)	Dominant	Semaglutide is cost-effective
Ericsson & Lundqvist 2017^80^ Sweden	T2DM	IDegLira vs other insulins	Markov model	5	40 years	Societal	3%	ITC	TTO	Many ICERs	IDegLira is estimated to be cost-effective
Escobar *et al.* 2022^81^ Spain	T2DM	Dapagliflozin vs SOC	Discrete Event Simulation model	2	30 years	Health care payer	3%	Clinical trial (DECLARE-TIMI 58)	EQ-5D	Dominant	Dapagliflozin would be cost-effective as an add-on therapy to SOC, compared with placebo
Evans *et al.* 2014^82^ UK	T2DM	Insulin degludec vs insulin glargine	No specification	14	1 year	Health care payer	No discounting	Clinical trial	EQ-5D	£13,078/QALY	Insulin degludec is a cost-effective treatment option compared with insulin glargine
Evans *et al.* 2015^83^ UK	T1DM	Insulin degludec vs insulin glargine	No specification	14	1 year	Health care payer	No discounting	Clinical trial	Unknown	£16,895/QALY	Insulin degludec is a cost-effective treatment option compared with insulin glargine
Evans *et al.* 2016^84^ Denmark	T2DM	Insulin degludec/insulin aspart (IDegAsp) vs BIAsp 30	No specification	18	5 years	Health care payer	3%	Clinical trials, open-label	EQ-5D	DKK 81,507/QALY	IDegAsp is a cost-effective treatment compared with BIAsp 30
Evans *et al.* 2017^85^ UK	T1DM and T2DM	Insulin degludec vs insulin glargine	Markov model	14	1 year	Health care payer	No discounting	Not reported	Unknown	Dominant	Insulin deludec is a cost-effective alternative to insulin glargine U100 for patients with diabetes
Evans *et al.* 2018^86^ UK	T1DM and T2DM	Insulin degludec vs insulin glargine	No specification	14	1 year	Health care payer	No discounting	Clinical trial (SWITCH 1 and SWITCH 2)	Unknown	T1DM: £984/QALY T2DM: £17,939/QALY	Insulin degludec is cost-effective
Evans *et al.* 2020^87^ Netherlands	T2DM	Insulin degludec vs insulin glargine	No specification	14	1 year	Societal	No discounting	Clinical trial (CONCLUDE)	EQ-5D	Dominant	Insulin degludec was a cost-effective relative to insulin glargine U300
Evans *et al.* 2023^88^ UK	T2DM	Semaglutide vs insulin aspart	Simulation model	3	Lifetime (50 years)	Health care payer	3.5%	Clinical trial (SUSTAIN-11)	EQ-5D	£4457/QALY	Semaglutide 1 mg highly cost-effective vs insulin aspart
Fonseca *et al.* 2013^89^ Spain	T2DM	Exenatide vs insulin glargine	Simulation model	3	35 years	Health care payer	3%	Clinical trial (DURATION 3 + pooled DURATION 1 and 5)	Unknown	€12,084/QALY	Exenatide is cost-effective
Franch-Nadal *et al.* 2022^90^ Spain	T2DM	Semaglutide vs empagliflozin, sitagliptin, liraglutide	Simulation model	3	Lifetime (50 years)	Health care payer	3%	Clinical trial (PIONEER 2, 3 & 4)	EQ-5D	Several ICERs	Oral semaglutide 14 mg dominant vs sitagliptin and liraglutide, and cost-effective vs empagliflozin
Gaebler *et al.* 2012^91^ US	T2DM	Exenatide vs pioglitazone, insulin	Simulation model	11	20 years	Health care payer	Unknown	Clinical trials	Unknown	Several ICERs	Exenatide dominant
Gæde *et al.* 2019^92^ Denmark	T2DM	Semaglutide vs liraglutide, exenatide, lixisenatide	Simulation model	3	Lifetime	Health care payer	3%	Clinical trial (SUSTAIN) and NMA	EQ-5D	Dominant	Semaglutide cost-effective alternative to other GLP-1 RA therapies
Gao *et al.* 2012^93^ China	T2DM	Liraglutide vs glimepiride	Simulation model	4	30 years	Health care payer	3%	Clinical trial (Yang *et al.* 2011)	EQ-5D	CNY 256,871/QALY	Liraglutide would be cost-effective
Goh *et al.* 2015^94^ Singapore	T2DM	Biphasic human insulin vs BIAsp 30	Simulation model	3	Lifetime	Health care payer and societal	3%	Clinical trial	EQ-5D	Dominant	BIAsp 30 is both a clinically effective and cost-saving treatment
Goodall *et al.* 2011^95^ Spain	T2DM	Exenatide vs insulin glargine	Simulation model	3	35 years	Health care payer	3%	Clinical trial	EQ-5D	€15,068/QALY	Exenatide represents an efficient option in comparison with insulin glargine
Gordon *et al.* 2016a^96^ UK	T2DM	Alogliptin vs sulfonylurea	Simulation model	3	Lifetime (50 years)	Health care payer	3.5%	Clinical trial (ENDURE)	EQ-5D	£10,959/QALY	Alogliptin is a cost-effective treatment alternative
Gordon *et al.* 2016b^97^ Sweden	T2DM	Exenatide vs insulin lispro	Simulation model	2	40 years	Health care payer	3%	Clinical trial (4B-study)	EQ-5D	€1971/QALY	Exenatide represents a cost-effective treatment
Gordon *et al.* 2017^98^ N/A	T2DM	Metformin + sulfonylurea vs metformin + TZD vs metformin + DPP-4 inhibitors	Simulation model	3	Lifetime (50 years)	Health care payer	3.5%	Observational study	EQ-5D	Several ICERs	Metformin + DPP-4 inhibitor treatment was associated with the largest gain in health benefit, and cost-effectiveness ratios were favorable
Gorgojo-Martínez *et al.* 2020^99^ Spain	T2DM	Semaglutide vs empagliflozin	Simulation model	3	Lifetime	Health care payer	3%	NMA	EQ-5D	Several ICERs	Semaglutide is cost-effective
Gourzoulidis *et al.* 2018^100^ Greece	T2DM	Empagliflozin vs SOC	Simulation model	18	Lifetime	Health care payer	3.5%	Clinical trial(EMPA-REG OUTCOME)	EQ-5D	€4633/QALY	Empagliflozin added to SOC was estimated to be highly cost-effective
Gourzoulidis *et al.* 2021^101^ Greece	T2DM	Empagliflozin vs dapagliflozin	Discrete event simulation model	12	Lifetime	Health care payer	3.5%	Clinical trial (EMPA-REG OUTCOME, DECLARE-TIMI 58)	EQ-5D	€965/QALY	Empagliflozin highly cost-effective compared to dapagliflozin
Granström *et al.* 2012^102^ Sweden	T2DM	Saxagliptin vs sulfonylurea	Simulation model	2	Lifetime	Health care payer	3%	Clinical trial (Göke *et al.*)	EQ-5D	SEK 9500/QALY	Saxagliptin is cost-effective
Grzeszczak *et al.* 2012^103^ Poland	T2DM	Saxagliptin + metformin/sulfonylurea vs NPH insulin	Simulation model	2	40 years	Health care payer	Costs: 5% Health-related effects: 3.5%	Clinical trial, meta-analysis	EQ-5D	PLN 27,454/QALY	Saxagliptin in combination with metformin or sulfonylurea is likely to represent a cost-effective treatment option
Guillermin *et al.* 2012^104^ US	T2DM	Exenatide vs sitagliptin or pioglitazone	Simulation model	3	Lifetime (35 years)	Health care payer	3%	Clinical trial (DURATION-2[ITT])	EQ-5D	Dominant	Exenatide is dominant
Gupta *et al.* 2015^105^ India, Indonesia, Saudi Arabia	T2DM	BIAsp 30 vs biphasic human insulin 30, insulin glargine, or NPH insulin	Simulation model	3	30 years	Not reported	Not reported	A1chieve study	EQ-5D	Several ICERs	BIAsp 30 is cost-effective
Gu *et al.* 2015^106^ China	T2DM	Saxagliptin vs glimepiride	Simulation model	2	40 years	Health care payer	3%	Head-to-head study (Zhu and Song)	EQ-5D	Dominant	Saxagliptin and metformin more cost-effective compared with glimepiride and metformin
Gu *et al.* 2016a^107^ China	T2DM	Dapagliflozin vs acarbose	Simulation model	2	40 years	Health care payer	3%	ITC	EQ-5D	Dominant	Dapagliflozin is dominant compared with acarbose
Gu *et al.* 2016b^108^ China	T2DM	Saxagliptin vs acarbose	Simulation model	2	Lifetime	Health care payer	3%	Meta-analysis (5 H2H trials)	EQ-5D	Dominant	SAXA + metformin is cost-effective compared with acarbose + metformin
Gu *et al.* 2017^109^ China	T2DM	Exenatide vs insulin glargine	Simulation model	2	40 years	Health care payer	3%	Meta-analysis	EQ-5D	Dominant	Exenatide twice daily is cost-effective
Gu *et al.* 2020^110^ China	T2DM	Metformin, sulfonylurea, TZD, alpha-glucosidase inhibitor, glinide, DPP-4 inhibitor, insulin, GLP-1 RA	Simulation model	2	40 years	Health care payer	3%	ITC	EQ-5D	Several ICERs	N/A
Guzauskas *et al.* 2021^111^ US	T2DM	Semaglutide vs sitagliptin, empagliflozin, liraglutide	Microsimulation	4	Lifetime	Health care payer	3%	NMA + clinical trial (PIONEER)	HUI3, EQ-5D	Several ICERs	Semaglutide cost-effective compared to sitagliptin, liraglutide but not compared to empagliflozin
Haig *et al.* 2016^112^ Canada	DME	Ranibizumab vs laser	Markov model	18	Lifetime	Health care payer and societal	5%	Clinical trial (RESTORE)	TTO, HUI	Health care: dominant Societal: C$24,494/QALY	Ranibizumab monotherapy and combination therapy provided greater benefits at lower costs than laser monotherapy (ranibizumab therapy dominated laser therapy)
Haldrup *et al.* 2020^113^ Italy	T1DM and T2DM	Degludec vs basal insulin	Simulation model	3	Max. 30 years	Health care payer	3%	Observational study (EU-TREAT)	EQ-5D	Dominant	Switching to degludec is dominant
Han *et al.* 2022^114^ China	T2DM	IDegLira vs insulin degludec/ liraglutide	Simulation model	4	40 years	Health care payer	5%	6 RCTs	EQ-5D	IDegLira: $99,464.12/QALY Insulin degludec/liraglutide: $143,348.26/QALY	IDegLira was not cost-effective
Holekamp *et al.* 2020^115^ US	DME	Aflibercept vs ranibizumab	Markov model	18	10 years	Health care payer	3%	Clinical trial (Protocol-T)	TTO	$711,301/QALY	Aflibercept is not cost-effective vs ranibizumab
Home *et al.* 2015^116^ Mexico, South Korea, India, Indonesia, and Algeria	T2DM	Insulin detemir vs SOC	Simulation model	3	30 years	Health care payer	3%	Observational study (A1chieve)	EQ-5D	Several ICERs	Starting insulin detemir cost-effective
Hou *et al.* 2019^117^ China	T2DM	Canagliflozin vs dapagliflozin	Simulation model	6	Lifetime	Health care payer	5%	NMA	EQ-5D	Dominant	The canagliflozin 100 mg strategy was a dominant option
Huang *et al.* 2022^118^ China	T2DM	Dapagliflozin + SOC vs SOC	Markov model	18	30 years	Health care payer	5%	Clinical trial (DECLARE-TIMI 58)	EQ-5D	€17,742.07/QALY	Dapagliflozin + standard treatment is a cost-effective option
Huetson *et al.* 2015^119^ Norway	T2DM	Lixisenatide vs insulin	Simulation model	3	Lifetime	Societal	4%	Indirect comparison	EQ-5D	Dominant	Lixisenatide may be considered an economically efficient therapy
Hunt *et al.* 2017a^120^ Netherlands	T2DM	IDegLira vs insulin glargine + insulin aspart	Simulation model	3	Lifetime (50 years)	Health care payer	1.5% and 5%	Clinical trial (DUAL-2)	Unknown	Dominant	This analysis suggests that IDegLira is cost-effective vs basal-bolus therapy
Hunt *et al.* 2017b^121^ Italy	T2DM	Liraglutide vs lixisenatide	Simulation model	3	20 years	Health care payer	3%	Clinical trial (open-label LIRA-LIXI trial)	EQ-5D	€2001/QALY	Liraglutide 1.8 mg is likely cost-effective compared with lixisenatide
Hunt *et al.* 2017c^122^ US	T2DM	IDegLira vs liraglutide	Simulation model	3	Lifetime (50 years)	Health care payer	3%	Indirect comparison	Unknown	Dominant	IDegLira was dominant vs liraglutide
Hunt *et al.* 2017d^123^ US	T2DM	IDegLira vs insulin glargine	Simulation model	3	20 years	Health care payer	3%	Clinical trial (DUAL-5)	EQ-5D	$63,678/QALY	IDegLira is cost-effective
Hunt *et al.* 2017e^124^ UK	T2DM	Liraglutide vs lixisenatide	Simulation model	3	20 years	Health care payer	3.5%	Clinical trial (open-label LIRA-LIXI trial)	Unknown	£8901/QALY	Liraglutide 1.8 mg likely to be highly cost-effective compared with lixisenatide 20 μg
Hunt *et al.* 2017f^125^ UK	T2DM	Liraglutide vs exenatide and lixisenatide	Simulation model	3	Lifetime (50 years)	Health care payer	3.5%	NMA	EQ-5D	Several ICERs	Liraglutide 1.2 mg is likely to be considered cost-effective vs alternative GLP-1 RAs
Hunt *et al.* 2019^126^ Netherlands	T2DM	Semaglutide vs insulin glargine vs dulaglutide	Simulation model	3	Lifetime	Societal	1.5% and 4%	Clinical trial (SUSTAIN-4 and SUSTAIN-7)	EQ-5D	Insulin glargine: €4988/QALY Dulaglutide: €495/QALY	Semaglutide is cost-effective vs insulin glargine U100, and dominant vs dulaglutide
Hu *et al.* 2021a^127^ China	T2DM	Dapagliflozin vs saxagliptin	Simulation model	5	Lifetime	Health care payer	5%	Meta-analysis (5 RCTs)	EQ-5D	$12,191.97/QALY	Dapagliflozin appears to be cost-effective therapy
Hu *et al.* 2021b^128^ China	T2DM	Semaglutide vs empagliflozin	Simulation model	4	40 years	Health care payer	5%	Meta-analysis (Lingway *et al.*)	EQ-5D	N/A	N/A
Hu *et al.* 2022a^129^ China	T2DM	Semaglutide vs dulaglutide	Simulation model	4	40 years	Health care payer	5%	Clinical trial (SUSTAIN-7)	EQ-5D	$26,957.44/QALY	Semaglutide expected to be cost-effective compared with dulaglutide
Hu *et al.* 2022b^130^ China	T2DM	Semaglutide vs dulaglutide, exenatide	Simulation model	4	40 years	Health care payer	5%	Meta-analysis	EQ-5D	Several ICERs	Dulaglutide appears to be the most cost-effective option among subcutaneous semaglutide
Hutton *et al.* 2019^131^ US	DR	Ranibizumab vs panretinal photocoagulation	Simulation model	18	10 years	Health care payer	3%	A preplanned secondary analysis of the Protocol S randomized clinical trial	TTO	$742,202/QALY	Ranibizumab compared with panretinal photocoagulation may be cost-effective for eyes presenting with proliferative DR and vision-impairing center-involved DME, but not for those with proliferative DR without vision-impairing center-involved-DME
Iannazzo *et al.* 2017^132^ Italy	T2DM	Empagliflozin + SOC vs SOC	Simulation model	18	Lifetime	Health care payer	3.5%	Clinical trial (EMPA REG OUTCOME)	EQ-5D	€4811/QALY	Empagliflozin in add-on to the SOC is a highly cost-effective strategy
Igarashi *et al.* 2022^133^ Japan	T2DM	SGLT2 inhibitors vs SOC	Microsimulation	18	10 years	Health care payer	2%	Observational study (CVD-REAL 2 study) + claims database	EQ-5D	Dominant	SGLT2 inhibitors can improve the clinical outcome and reduce cost burden of T2DM
Ishii *et al.* 2018^134^ Japan	T2DM	Dulaglutide vs insulin glargine	Simulation model	3	Lifetime (50 years)	Health care payer	2%	Clinical trial (Araki 2015 trial)	EQ-5D	JPY 416,280/QALY	Dulaglutide 0.75 mg may be cost-effective
Jendle *et al.* 2020^135^ Sweden	T1DM and T2DM	Insulin degludec vs other basal insulins	Simulation model	3	Lifetime	Societal	Unknown	Observational study (ReFLeCT)	EQ-5D	Several ICERs	Insulin degludec was cost-effective or dominant vs other basal insulins
Johansen *et al.* 2019^136^ Canada	T2DM	Semaglutide vs dulaglutide	Markov model	5	40 years	Societal	1,5%	Clinical trial (SUSTAIN-7)	EQ-5D (Bagust & Beale)	Dominant	Semaglutide may be cost-efective
Johansen *et al.* 2020^137^ UK	T2DM	Semaglutide vs liraglutide	Simulation model	3	Lifetime (50 years)	Health care payer	3.5%	Clinical trial (SUSTAIN-10)	EQ-5D	Dominant	Once-weekly semaglutide is a cost-effective treatment
Kaku *et al.* 2019^138^ Japan	T2DM	Empagliflozin + SOC vs SOC	Discrete event simulation model	12	Lifetime	Health care payer	2%	Clinical trial (EMPA REG OUTCOME)	EQ-5D	415,849 yen/QALY	Empagliflozin added to SOC is highly cost-effective compared with SOC alone
Kansal *et al.* 2019^139^ UK	T2DM	Empagliflozin + SOC vs SOC	Discrete event simulation model	12	3 years	Health care payer	3.5%	Clinical trial (EMPA REG OUTCOME)	EQ-5D	£4083/QALY	Empagliflozin in addition to SOC is projected to be highly cost-effective
Kiadaliri *et al.* 2014^140^ Sweden	T2DM	GLP-1 vs DPP-4, NPH insulin	Markov model	5	35 years	Societal	3%	Observational study	EQ-5D	Several ICERs	GLP-1 RA is cost-effective
Klarenbach *et al.* 2011^141^ Canada	T2DM	Metformin vs sulfonylureas, DPP-4 inhibitors, TZDs	Simulation model	4	40 years	Health care payer	5%	SLR + mixed treatment comparison meta-analysis	EQ-5D	Several ICERs	Sulphonylurea the most cost-effective
Kourlaba *et al.* 2016^142^ Greece	DME	Ranibizumab vs aflibercept	Markov model	18	Lifetime	Health care payer	3.5%	NMAs, clinical trials	TTO	Dominant	Ranibizumab dominant
Kousoulakou *et al.* 2017^143^ Greece	T2DM	Vildagliptin vs glimepiride	Simulation model	13	Lifetime	Health care payer	3,5%	Head-to-head trial, + meta-analysis	EQ-5D	Dominant	Vildagliptin as add-on treatment to metformin dominant vs glimepiride
Krishnamoorthy *et al.* 2022^144^ India	T2DM	Dapagliflozin vs canagliflozin	Decision tree	18	Unknown	Health care payer	Unknown	Observational study	Unknown	N/A	N/A
Kvapil *et al.* 2017^145^ Czech Republic	T2DM	IDegLira vs insulin	Simulation model	3	Lifetime	Health care payer	3%	Clinical trial (DUAL)	Unknown	CZK 345,052/QALY	IDegLira is cost-effective
Lalic *et al.* 2018^146^ Serbia	T1DM and T2DM	Insulin degludec vs insulin glargine	Markov model	14	1 year	Health care payer	No discounting	Meta-analysis	Unknown	T1DM: RSD 417,586/QALY, T2DM: RSD 558,811/QALY	Degludec is a cost-effective alternative to glargine
Langer *et al.* 2019^147^ Japan	T2DM	Insulin degludec vs insulin	Markov model	14	1 year	Health care payer	No discounting	Observational study (The KIDUNA Study)	EQ-5D	JPY 268,811/QALY	Degludec is cost-effective
Landstedt-Hallin *et al.* 2017^148^ Sweden	T1DM	Insulin degludec vs insulin	Simulation model	3	Lifetime	Health care payer and societal	3%	Observational study	EQ-5D	Dominant	Insulin degludec is dominant
Lasalvia *et al.* 2017^149^ Colombia	T2DM	Dulaglutide vs liraglutide and glargine	Markov model	18	5 years	Health care payer	5%	Clinical trials	EQ-5D	Several ICERs	Dulaglutide dominates liraglutide. ICER compared with glargine higher than the accepted threshold
Lasalvia *et al.* 2022^150^ Colombia	T2DM	Dapagliflozin + metformin vs DPP-4 inhibitors + metformin	Simulation model	2	5 years	Health care payer	5%	Meta-analysis	EQ-5D	$1964.80/QALY gained	The combination of dapagliflozin with metformin is a cost-effective option compared to DPP-4 + metformin inhibitors
Lau *et al.* 2019^151^ Hong Kong	T2DM	Insulin glargine vs NPH	Simulation model	3	50 years	Societal	3%	Clinical trial (the LEAD study)	EQ-5D	HKD 98,663/QALY	Insulin glargine U100 is cost-effective
Lee *et al.* 2011^152^ US	T2DM	Liraglutide + glimepiride vs rosiglitazone + glimepiride	Simulation model	3	35 years	Health care payer	3%	Clinical trial (the LEAD study)	EQ-5D (Palmer *et al.*)	$34,147/QALY and $56,190/QALY	Liraglutide + glimepiride is cost-effective
Lee *et al.* 2012^153^ US	T2DM	Liraglutide vs sitagliptin	Simulation model	3	35 years	Health care payer	3%	Clinical trial (1860-LIRADPP-4 trial)	EQ-5D	$37,234/QALY	ICERs remained below a commonly accepted threshold in the US, except for the shortest time horizon of 10 years
Lin *et al.* 2018^154^ US	DME	Vitrectomy vs ranibizumab	No specification	18	Lifetime	Not reported	Not reported	Clinical trial	Unknown	$338,348/QALY	Vitrectomy is more advantageous
Lin *et al.* 2021^155^ China	T2DM	Different DPP-4-inhibitors vs each other	Simulation model	6	Lifetime	Health care payer	5%	SLR + mixed treatment comparison	EQ-5D	Several ICERs	Alogliptin was a preferred treatment option compared with other DPP-4 inhibitors
Lin *et al.* 2022^156^ US	T2DM	Tirzepatide vs semaglutide or empagliflozin	Microsimulation	4	Lifetime	Health care payer	3%	NMA	HUI3	N/A	N/A
Liu *et al.* 2022^157^ China	T2DM	Semaglutide vs polyethylene glycol loxenatide	Markov model	5	40 years	Health care payer	5%	NMA	EQ-5D (Pan *et al.*)	Dominant	Semaglutide a dominant option vs polyethylene glycol loxenatide
Luo *et al.* 2022^158^ China	T2DM	Insulin degludec/insulin aspart (IDegAsp) vs BIAsp 30	Simulation model	3	30 years	Health care payer	5%	Clinical trial (Yang *et al.*)	Unknown	CNY 13,886/QALY	Compared with BIAsp 30, IDegAsp was cost-effective
Malkin *et al.* 2019a^159^ Estonia	T2DM	Semaglutide vs liraglutide	Simulation model	3	Lifetime	Health care payer	5%	NMA (Witkovski *et al.*)	EQ-5D	€523/QALY	Semaglutide highly cost-effective vs liraglutide
Malkin *et al.* 2019b^160^ Slovakia	T2DM	Semaglutide vs dulaglutide	Simulation model	3	Lifetime	Health care payer	5%	Clinical trial (SUSTAIN-7)	EQ-5D	Dominant	Semaglutide representscost-saving treatment
Malkin *et al.* 2021^161^ Netherlands	T2DM	Semaglutide vs empagliflozin, sitagliptin, liraglutide	Simulation model	3	Lifetime	Health care payer	1.5% and 4%	Clinical trial (PIONEER 2, 3 and 4)	Unknown	Several ICERs	Semaglutide cost-effective vs empagliflozin and sitagliptin and dominant vs liraglutide
Malkin *et al.* 2022^162^ Portugal	T2DM	Semaglutide vs empagliflozin and dulaglutide	Simulation model	3	Lifetime (50 years)	Health care payer	4%	Clinical trial (PIONEER-2), NMA	EQ-5D	Empa: €23,571/QALY Dula: €23,927/QALY	Oral semaglutide 14 mg cost-effective
Martín *et al.* 2020^163^ Spain	T2DM	Semaglutide vs dulaglutide and sitagliptin	Simulation model	3	Lifetime	Health care payer	3%	Clinical trial (SUSTAIN 7 and 2)	EQ-5D	Dominant	Once-weekly semaglutide 0.5 mg and 1 mg were considered dominant
McCrimmon *et al.* 2021a^164^ UK	T2DM	iGlarLixi vs other insulins	Simulation model	3	Lifetime	Health care payer	3.5%	ITC (AWARD-9, LIRA ADD2 BASAL, DUAL-V	EQ-5D	Several ICERs	iGlarLixi provides very similar outcomes and substantial cost savings
McCrimmon *et al.* 2021b^165^ UK	T2DM	iGlarLixi vs iDegLira	Simulation model	3	Lifetime	Health care payer	3.5%	LixiLan-G study; Home *et al.*; GetGoal Duo-2 trial	EQ-5D	Dominant	iGlarLixi can be considered as cost-effective
McCrimmon *et al.* 2022^166^ UK	T2DM	iGlarLixi vs Premix BIAsp 30	Simulation model	3	Lifetime	Health care payer	3.5%	Clinical trial (SoliMix)	Unknown	£13,598 /QALY	iGlarLixi is a simple, cost-effective option
McEwan *et al.* 2020^167^ UK, US, China	T2DM	SGLT2 inhibitors vs SOC	Simulation model	2	Lifetime	Health care payer	Several rates	Observational study (CVD-REAL) and meta-analysis (Zelniker)	EQ-5D	N/A	N/A
McEwan *et al.* 2021^168^ UK	T2DM	Dapagliflozin vs SOC	Simulation model	2	Lifetime	Health care payer	3.5%	Clinical trial (DECLARE-TIMI 58)	EQ-5D	Dominant	Dapagliflozin compared to placebo appears to be cost-effective
Men *et al.* 2020a^169^ China	T2DM	Lixisenatide + insulin vs premix insulin	Simulation model	3	Lifetime	Health care payer	3%	Clinical trial (GetGoalL-C) + NMA	Unknown	Several ICERs	Lixisenatide is cost-effective
Men *et al.* 2020b^170^ China	T2DM	Lixisenatide vs insulin	Simulation model	3	Lifetime	Health care payer	3%	Mixed treatment comparison meta-analysis	Unknown	Several ICERs	Lixisenatide was also a cost-effective treatment
Men *et al.* 2020c^171^ China	T2DM	Empagliflozin + SOC vs SOC	Simulation model	12	Lifetime	Health care payer	3.5%	Clinical trial (EMPA REG OUTCOME)	EQ-5D	¥3988 /QALY	Empagliflozin added to best available standard care was estimated to be a highly value-for-money
Mezquita-Raya *et al.* 2013^172^ Spain	T2DM	Liraglutide vs sitagliptin	Simulation model	3	Lifetime	Health care payer	3%	Clinical trial (Pratley *et al.*)	EQ-5D (Davies *et al.* CEA)	€13,266/QALY	Liraglutide is likely to becost-effective vs sitagliptin
Mezquita-Raya *et al.* 2017a^173^ Spain	T1DM and T2DM	Insulin degludec vs insulin glargine	No specification	14	1 year	Health care payer	No discounting	6 RCTs	TTO	T1DM: €52.70/QALY T2DM: €11,240/QALY	Degludec is cost-effective
Mezquita-Raya *et al.* 2017b^174^ Spain	T2DM	Liraglutide vs lixisenatide	Simulation model	3	Lifetime	Health care payer	3%	Clinical trial (LIRA-LIXI trial)	Unknown	€4113 QALY	Liraglutide 1.8 mg is likely to be cost-effective compared with lixisenatide 20 mg
Ming *et al.* 2020^175^ China	DME	Aflibercept vs laser or ranibizumab	Markov model	18	20 years	Societal	5%	Clinical trial (VIVID-EAST) + ITC	TTO	Laser: CNY 129,396/QALY Ranibizumab: CNY –12,774/QALY	Intravitreal aflibercept cost-effective compared to laser and dominant compared to ranibizumab
Mitchell *et al.* 2012^176^ UK	DME	Ranibizumab (+ laser) vs laser	Markov model	10	15 years	Health care payer	3.5%	Clinical trial (RESTORE-trial)	EQ-5D	£24,028/QALY and £36,106/QALY	Ranibizumab monotherapy appears to be cost-effective relative to laser monotherapy; combination is less certain
Montes Rodríguez *et al.* 2022^177^ Spain	DME	Dexamethasone vs aflibercept	Markov model	18	Lifetime	Societal	3.5%	Clinical trial (Schwartz *et al.* trial + Vivid-East study)	TTO	€29.00/QALY	Dexamethasone may be cost-effective
Morales *et al.* 2015^178^ Spain	T1DM and T2DM	Insulin detemir vs NPH	No specification	15, 16	1 year	Health care payer	No discounting	Clinical trials and observational studies	Unknown	Several ICERs	Insulin detemir was cost-effective
Morton *et al.* 2022^179^ Australia	T2DM	SGLT2 inhibitors vs GLP-1 RA	Microsimulation	18	Unknown	Societal	5%	Meta-analyses	EQ-5D	Several ICERs	Use of SGLT2 inhibitors, but not GLP-1 RAs, would be cost-effective
Neslusan *et al.* 2015^180^ Mexico	T2DM	Canagliflozin vs sitagliptin	Microsimulation	6	20 years	Health care payer	5%	Clinical trial, NMA	EQ-5D (Bagust & Beale)	Several ICERs	Doses of canagliflozin are likely to be cost-effective
Neslusan *et al.* 2018^181^ US	T2DM	Canagliflozin vs dapagliflozin	Microsimulation	6	30 years	Health care payer	3%	NMA	EQ-5D	Dominant	Canagliflozin is dominant
Nguyen *et al.* 2018^182^ US	T2DM	Empagliflozin + SOC vs SOC	Markov model	18	Lifetime	Health care payer	3%	Clinical trial (EMPA REG OUTCOME)	Unknown	$76,167/QALY	Empagliflozin may be cost-effective compared to standard treatment
Nguyen-Thi *et al.* 2020^183^ Vietnam	T1DM	Gliclazide vs SOC	Partitioned-survival model	18	5 years	Health care payer	3%	Clinical trial (ADVANCE-study)	EQ-5D	$1878/QALY	Gliclazide-based intensive glucose control is cost-effective
Nian *et al.* 2020^184^ China	T2DM	Dapagliflozin vs metformin	Simulation model	6	Lifetime	Health care payer	5%	NMA	EQ-5D	Dominated	Dapagliflozin is not likely to be cost-effective compared with metformin
NICE 2013a^185^ UK	DME	Fluocinolone vs laser	Markov model	19	15 years	Health care payer	3.5%	Clinical trial	TTO	Several ICERs	Fluocinolone acetonide intravitreal implant was not recommended for treating chronic DME; a patient access scheme is needed
NICE 2013b^186^ UK	DME	Ranibizumab vs laser	Markov model	19	15 years	Health care payer	Unknown	Clinical trial	EQ-5D	N/A	Ranibizumab and laser photocoagulation as part of a simultaneous treatment strategy could not be recommended
NICE 2013c^187^ UK	T2DM	Dapagliflozin + insulin vs insulin and dapagliflozin + metformin vs metformin	Simulation model	19	40 years	Health care payer	3.5%	NMA	EQ-5D	Several ICERs	Dapagliflozin in combination with insulin with or without other antidiabetic drugs is recommended
NICE 2014^188^ UK	T2DM	Canagliflozin vs sulfonylureas, TZDs, DPP-4 inhibitors, GLP-1 analogs, dapagliflozin and insulin	Microsimulation	19	40 years	Health care payer	3.5%	NMA	EQ-5D	Several ICERs	Canagliflozin cost-effective combined with metformin, triple therapy in combination with metformin and either a sulfonylurea or a TZD, and as an add-on treatment to insulin
NICE 2015a^189^ UK	DME	Aflibercept vs ranibizumab/dexamethasone/fluocinolone/laser	Markov model	19	Unknown	Health care payer	Unknown	NMA, ITC, clinical trial	EQ-5D and TTO	Several ICERs	Aflibercept is recommended for treating visual impairment only if the eye has a central retinal thickness of 400 micrometres or more at the start of treatment and the company provides aflibercept with the discount agreed in the patient access scheme
NICE 2015b^190^ UK	T2DM	Empagliflozin vs dapagliflozin/canagliflozin/sitagliptin	Simulation model	19	Lifetime	Health care payer	3.5%	NMA	EQ-5D	Several ICERs	Empagliflozin in combination with metformin is recommended
NICE 2016^191^ UK	T2DM	Dapagliflozin vs SOC	Simulation model	19	40 years	Health care payer	3.5%	Clinical trial, NMA	EQ-5D	Several ICERs	Dapagliflozin in combination with insulin with or without other antidiabetic drugs is recommended
NICE 2020^192^ UK	T1DM	Sotagliflozin + SOC vs SOC	Simulation model	19	60 years	Health care payer	3.5%	Pooled clinical trials	EQ-5D	£1934/QALY	Recommendation to consider the use of sotagliflozin in overweight or obese T1DM patients
NICE 2022a^193^ UK	DME	Dexamethasone vs sham/ranibizumab/laser/luocinolone	Markov model	19	15 years	Health care payer	3.5%	NMA	VFQ-UI, National Eye Institute VFQ-25, and TTO	Several ICERs	Dexamethasone intravitreal implant is recommended for treating visual impairment only if their condition has not responded well enough to, or if they cannot have non-corticosteroid therapy
NICE 2022b^194^ UK	DME	Brolucizumab vs aflibercept or ranibizumab	Markov model	19	Lifetime	Health care payer	3.5%	NMA	VFQ-25	N/A	Brolucizumab is recommended for treating visual impairment, only if: the eye has a central retinal thickness of 400 micrometres or more at the start of treatment AND the company provides brolucizumab according to the commercial arrangement
Nilsson *et al.* 2022^195^ Sweden	T2DM	Empagliflozin vs SOC	Markov model	5	40 years	Health care payer and societal	3%	Clinical trial (EMPA REG OUTCOME)	EQ-5D (Beaudet *et al.*)	€16,000/QALY	A broader implementation of empagliflozin would lead to further benefits even from a short-term perspective
Nita *et al.* 2012^196^ Brazil	T2DM	Saxagliptine + metformin vs rosiglitazone or pioglitazone	Discrete event simulation model	4	3 years	Private health care payer	5%	SLR	EQ-5D	Dominant	Adding saxagliptine to metformin is cost-saving
Pakdaman *et al.* 2020^197^ Iran	T1DM and T2DM	Insulin analogs vs regular insulin	Decision tree	18	Unknown	Provider	Unknown	Patient records	EQ-5D	$0.093506/QALY	Insulin analogs are more cost-effective than regular insulin
Pawaskar *et al.* 2019a^198^ US	T2DM	SGLT2 inhibitors + DPP-4 inhibitors vs GLP-1	Simulation model	3	Lifetime	Health care payer	3%	RCT + meta-analysis	EQ-5D (Beaudet)	$64,784/QALY	SGLT2 inhibitors may be cost-effective
Pawaskar *et al.* 2019b^199^ UK	T2DM	SGLT2 inhibitor vs NPH insulin	Simulation model	3	Lifetime	Health care payer	3.5%	RCT	EQ-5D (Beaudet)	Dominant	SGLT2 inhibitor is cost-neutral or cost-effective
Pawaskar *et al.* 2021^200^ US	T2DM	SGLT2 inhibitor vs GLP-1 RA	Simulation model	3	Lifetime	Health care payer	3%	RCT	EQ-5D (Beaudet)	Dominant	SGLT2 inhibitor on top of a DPP-4 inhibitor demonstrated slightly better efficacy and cost savings compared with switching to a GLP-1 RA
Peng *et al.* 2022^201^ Taiwan	T2DM	SGLT2 inhibitor vs DPP-4 inhibitor	Markov model	18	10 years	Health care payer	3%	A retrospective cohort study (Yang *et al.*)	EQ-5D (Kuo *et al.*)	$3244	SGLT2 inhibitor vs DPP-4 inhibitor was highly cost-effective
Pérez *et al.* 2015^202^ Spain	T2DM	Liraglutide vs sitagliptin	Simulation model	3	Lifetime	Health care payer	3%	Clinical trial (The 1860-LIRA-DPP-4 trial)	Unknown	€10,436/QALY	Liraglutide is cost-effective
Permsuwan *et al.* 2016a^203^ Thailand	T2DM	Insulin glargine vs NPH insulin	Simulation model	3	Lifetime	Health care payer	3%	Meta-analysis + TITAN Program	Unknown	THB 244,915/QALY	Insulin glargine is not cost-effective
Permsuwan *et al.* 2016b^204^ Thailand	T2DM	DPP-4-inhibitors vs metformin and sulfonylurea	Simulation model	3	Lifetime	Health care payer	3%	Meta-analysis (Wu *et al.*)	Unknown	$110,133.50/QALY	DPP-4 inhibitors were not cost-effective
Permsuwan *et al.* 2017^205^ Thailand	T2DM	Insulin detemir vs insulin glargine	Simulation model	3	50 years	Health care payer	3%	Observational study (TITAN Program) + meta-analysis (Swinnen et al) + CADTH assessment	Unknown	$1.7 million/QALY	Insulin detemir not cost-effective
Pershing *et al.* 2014^206^ US	DME	Laser treatment, triamcinolone or a vascular endothelial growth factor inhibitor	Markov model	18	Lifetime	Societal	3%	Clinical trials (RESTORE, RISE, RIDE, READ-2, RESOLVE, ETDRS)	TTO	Several ICERs	The most effective treatment is vascular endothelial growth factor inhibitor injections with or without laser treatment
Pesonen *et al.* 2021^207^ Finland	DME	Dexamethasone vs triamcinolone	Markov model	18	2 year and 5 year	Health care payer	3%	Clinical & observational studies	TTO	2-year: €56,243/QALY 5-year: dominated	Dexamethasone cost-effective during the first 2 years with willingness-to-pay threshold around €55000/QALY, and triamcinolone would be a convenient treatment after that
Pfohl *et al.* 2012^208^ Germany	T1DM	Insulin glargine vs NPH insulin	Discrete event simulation model	9	40 years	Health care payer	3%	Clinical and observational studies	EQ-5D	Dominant	Insulin glargine cost- effective or even cost-saving
Pochopien *et al.* 2019^209^ UK	DME	Fluocinolone acetonide vs dexamethasone or SOC	Markov model	18	15 years	Health care payer	3.5%	Clinical trial (FAME) + NMA	EQ-5D	SOC: £16,609/QALY Dexamethasone: £14,070/QALY	The fluocinolone acetonide 0.2 μg/day implant provided good value for money
Pöhlmann *et al.* 2019a^210^ Italy	T2DM	IDegLira vs IGlarLixi	Simulation model	3	Lifetime	Health care payer	3%	ITC (Evans *et al.*)	EQ-5D	€7386/QALY	IDegLira cost-effective vs iGlarLixi
Pöhlmann *et al.* 2019b^211^ Czech Republic	T2DM	IDegLira vs IGlarLixi	Simulation model	3	Lifetime	Health care payer	3%	ITC (Buse *et al.*)	EQ-5D	CZK 695,998/QALY	IDegLira was likely to be cost-effective
Pollock *et al.* 2011^212^ Japan	T2DM	Insulin aspart vs human insulin	Discrete event simulation model	18	5 and 10 years	Health care payer	3%	Clinical trial (The Nippon Ultra-Rapid Insulin and Diabetic Complication Evaluation-Study)	EQ-5D	Dominant	Insulin aspart resulted in increased quality of life and decreased costs when compared with human insulin
Pollock *et al.* 2012a^213^ UK	T2DM	75/25 biphasic insulin lispro and 50/50 biphasic insulin lispro vs long-acting insulin	Simulation model	3	35 years	Health care payer	3.5%	Meta-analysis (Qayyum *et al.*)	Unknown	75/25: £1217/QALY 50/50: £430/QALY	Biphasic analog insulins are likely to improve clinical outcomes and reduce costs
Pollock *et al.* 2012b^214^ US	T2DM	75/25 biphasic insulin lispro and 50/50 biphasic insulin lispro vs long-acting insulin	Simulation model	3	35 years	Health care payer	3%	Meta-analysis (Qayyum *et al.*)	Unknown	75/25: $1724/QALY 50/50: $1720/QALY	Biphasic analog insulins are likely to improve clinical outcomes and reduce costs
Pollock *et al.* 2018a^215^ UK	T1DM and T2DM	Insulin detemir vs NPH insulin	No specification	18	1 year	Health care payer	No discounting	Meta-analysis	EQ-5D + TTO	£610/QALY	Insulin detemir is a cost-effective alternative
Pollock *et al.* 2018b^216^ UK	T2DM	Insulin degludec vs insulin glargine	Simulation model	18	2 years	Health care payer	3.5%	Clinical trial (DEVOTE)	EQ-5D (Clarke *et al.*)	Dominant	Insulin degludec was cost-neutral
Pollock *et al.* 2019a^217^ Canada	T2DM	Insulin degludec vs insulin glargine	No specification	18	2 years	Health care payer	1.5%	Clinical trial (DEVOTE trial)	EQ-5D (Clarke *et al.*)	Dominant	Insulin degludec improved clinical outcomes at a lower cost as compared to glargine
Pollock *et al.* 2019b^218^ Canada	T2DM	Dulaglutide vs insulin glargine	Simulation model	18	40 years	Health care payer	1.5%	Clinical trial (AWARD-2)	EQ-5D	C$52,580	Dulaglutide 1.5 mg would likely be cost-effective
Pollock *et al.* 2019c^219^ UK	T2DM	Insulin degludec vs insulin glargine	Microsimulation	4	40 years	Health care payer	3.5%	Clinical trial (DEVOTE-CV)	EQ-5D	£14,956/QALY	Degludec was cost-effective
Pollock & Tikkanen 2016^220^ Denmark	T1DM and T2DM	Insulin degludec vs insulin glargine	No specification	14	1 year	Health care payer	No discounting	Meta-analyses, other studies	TTO	T1DM: Dominant T2DM: DKK 221,063/QALY	Insulin degludec resulted in cost savings relative to insulin glargine in T1D and T2D basal-only therapy cohorts, while being cost-effective in T2D basal-bolus therapy
Psota *et al.* 2017^221^ Slovakia	T2DM	IDegLira vs basal-bolus insulin	Simulation model	3	Lifetime (50 years)	Health care payer	5%	Pooled analysis (Freemantle *et al.*)	Unknown	€8590/QALY	IDegLira is cost-effective
Ramos *et al.* 2019^222^ UK	T2DM	Empagliflozin vs sitagliptin or saxagliptin	Simulation model	3	50 years	Health care payer	3.5%	Clinical trials (EMPA-REG OUTCOME, TECOS and SAVOR-TIMI 53)	EQ-5D	Several ICERs	Empagliflozin is cost-effective
Ramos *et al.* 2020a^223^ UK	T2DM	Empagliflozin vs oral semaglutide	Simulation model	3	50 years	Health care payer	3.5%	Clinical trial (PIONEER-2) + observational study (EMPRISE)	EQ-5D	Dominant	Empagliflozin is a cost-effective treatment option vs oral semaglutide
Ramos *et al.* 2020b^224^ UK	T2DM	Empagliflozin vs liraglutide	Simulation model	3	50 years	Health care payer	3.5%	Clinical trial (EMPA REG OUTCOME + LEADER)	EQ-5D	Dominant	Empagliflozin+ SOC is cost-effective
Ramos *et al.* 2021^225^ China	T2DM	Empagliflozin vs sitagliptin or liraglutide	Simulation model	3	Lifetime	Health care payer	3%	Clinical trial (EMPA REG OUTCOME) + ITC	EQ-5D	Several ICERs	Empagliflozin+ SOC is cost-effective
Raya *et al.* 2019^226^ Spain	T2DM	IDegLira vs insulin or GLP-1	Simulation model	3	50 years	Health care payer	3%	Observational study (EXTRA)	Unknown	Several ICERs	IDegLira is likely to improve clinical outcomes and reduce costs or be cost-effective
Régnier *et al.* 2015^227^ UK	DME	Ranibizumab vs aflibercept	Markov model	10	Lifetime	Health care payer	3.5%	NMA (Régnier *et al.*)	TTO	Dominant	Ranibizumab provides greater health gains with lower overall costs than aflibercept
Reifsnider *et al.* 2021a^228^ US	T2DM	Empagliflozin vs sitagliptin	Simulation model	18	Lifetime	Health care payer	3%	ITC, NMA	EQ-5D	With CVD: $3589/QALY, without CVD $12,577/QALY	Empagliflozin was cost-effective
Reifsnider *et al.* 2021b^229^ US	T2DM	Empagliflozin vs canagliflozin, dapagliflozin, or SOC	Discrete event simulation model	18	Lifetime	Health care payer	3%	ITC	EQ-5D	Several ICERs	Empagliflozin dominate canagliflozin and highly cost-effective compared with dapagliflozin
Reifsnider *et al.* 2022^230^ US	T2DM	Empagliflozin vs liraglutide	Simulation model	18	Lifetime	Health care payer	3%	NMA	EQ-5D	Dominant	Empagliflozin combined with metformin dominant
Ridderstråle *et al.* 2013^231^ Denmark, Norway, Sweden, Finland	T2DM	Insulin detemir vs NPH insulin	No specification	15	1 year	Health care payer	No discounting	RCT	EQ-5D	Several ICERs	Insulin detemir compared with NPH insulin provide economic benefits in the short-term
Rind *et al.* 2019^232^ US	T2DM	Oral semaglutide vs empagliflozin, sitagliptin and no treatment	Microsimulation	4	Lifetime	Health care payer	3%	12 RCTs	HUI3	Several ICERs	Semaglutide is cost-effective compared to liraglutide and sitagliptin, but not compared to empagliflozin
Risebrough *et al.* 2021^233^ US	T2DM	Oral semaglutide vs dulaglutide or liraglutide	Markov model	18	Lifetime	Health care payer	3%	NMA	HUI3, EQ-5D	Several ICERs	Oral semaglutide is a cost-effective GLP-1 RA treatment option
Rojas & Nunes 2021^234^ Ecuador	T2DM	Sitagliptin/metformin vs glibenclamid/metformin	Decision tree	18	Unknown	Health care payer	Not reported	Meta-analysis	Unknown	$611.11/QALY	Metformin/sitagliptin compared to metformin /glibenclamide was shown highly cost-effective in the public health system
Ross *et al.* 2016^235^ US	DME	Aflibercept, bevacizumab, and ranibizumab	No specification	18	10 years	Health care payer	3%	Post-hoc clinical trial	TTO	Several ICERs	Aflibercept (2.0 mg) and ranibizumab (0.3 mg) are not cost-effective relative to bevacizumab
Roussel *et al.* 2016^236^ France	T2DM	Liraglutide vs sitagliptin or glimepiride	Simulation model	3	Lifetime	Health care payer	3%	Clinical trial (LIRA-DPP4 and LEAD-2)	Unknown	Sitagliptin: €10,275/QALY Glimepiride: €20,709/QALY	Liraglutide is likely to be cost-effective
Ruan *et al.* 2022^237^ China	T2DM	Semaglutide vs dulaglutide	Semi-Markov	5	40 years	Health care payer	5%	Clinical trials	EQ-5D (Clarke *et al.*)	Dominant	QW semaglutide dominant
Russel-Jones *et al.* 2017^238^ UK	T1DM	Fast-acting insulin aspart vs conventional insulin aspart	Simulation model	3	70 years	Health care payer	3.5%	Clinical trial (Russel-Jones *et al.*)	Unknown	Dominant	Faster aspart was associated with improved clinical outcomes and cost savings
Russel-Szymczyk *et al.* 2019^239^ Bulgaria	T1DM and T2DM	Insulin degludec vs insulin glargine	No specification	18	1 year	Health care payer	No discounting	Multi-national survey + observational study	Unknown	Several ICERs	Degludec is a cost-effective alternative
Sabale *et al.* 2015^240^ Denmark, Norway, Sweden, Finland	T2DM	Dapagliflozin vs sulfonylureas	Simulation model	2	Lifetime	Health care payer	3%	Clinical trial (Nauck *et al.*)	EQ-5D	Several ICERs	Dapagliflozin cost-effective.
Sabapathy *et al.* 2016^241^ Canada	T2DM	Canagliflozin vs sulfonylurea	Microsimulation	7	40 years	Health care payer	5%	Head-to-head trial (Nicolle *et al.*)	Unknown	Dominant	Canagliflozin provided better health outcomes and lower costs
Salem *et al.* 2021^242^ China	T2DM	Empagliflozin vs glimepiride	Simulation model	3	50 years	Health care payer	5%	Clinical trials (EMPA-REG H2H-SU trial)	EQ-5D (Li *et al.*)	$4364/QALY	Empagliflozin is cost-effective treatment
Samyshkin *et al.* 2012^243^ US	T2DM	Exenatide vs insulin glargine	Simulation model	3	Lifetime	Health care payer	3%	Clinical trial (DURATION-3)	EQ-5D (Clarke *et al.*)	$15,936/QALY	Treatment with exenatide QW is cost-effective
Shafie *et al.* 2014^244^ Saudi Arabia, India, Indonesia, and Algeria	T2DM	BIAsp 30 vs SOC	Simulation model	3	30 years	Health care payer	3%	Clinical trial	EQ-5D	Several ICERs	BIAsp 30 is cost-effective
Shafie & Ng 2020^245^ Malaysia	T2DM	Insulin glargine or insulin detemir vs NPH insulin	Simulation model	4	40 years	Health care payer	3%	Literature review	EQ-5D (Default UKPDS-OM2)	Insulin grargine: $2835/QALY Insulin detemir: dominant	Both insulin detemir and glargine are cost-effective compared to NPH insulin
Shah *et al.* 2018^246^ US	T2DM	Liraglutide + SOC vs SOC	Markov model	18	Lifetime (30 years)	Health care payer	3%	Clinical trial (LEADER)	EQ-5D	$106,749/QALY	Liraglutide is cost-effective
Shao *et al.* 2017^247^ China	T2DM	Dapagliflozin vs glimepiride	Simulation model	2	40 years	Health care payer	3%	Indirect comparison, meta-analysis	EQ-5D	Dominant	Dapagliflozin is cost-effective
Shao *et al.* 2022^248^ US	T2DM	iGlarLixi vs Premix BIAsp 30	Simulation model	3	Lifetime	Health care payer	3%	Clinical trial (SoliMix)	EQ-5D (Beaudet *et al.* 2014)	Dominant	iGlar-Lixi confers improved QALYs and reduced costs compared with BIAsp 30
Smith-Palmer *et al.* 2012^249^ Sweden	T2DM	Insulin detemir vs NPH insulin	Simulation model	3	40 years	Health care payer	3%	RCT	EQ-5D (Default UKPDS-OM2)	Dominant	It is likely that insulin detemir would be cost-saving in comparison with NPH insulin
Stafford *et al.* 2022^250^ Canada	T2DM	Semaglutide vs canagliflozin	Simulation model	5, 7	40 years	Health care payer and societal	1.5%	RCT (SUSTAIN 8)	EQ-5D (Code-2 study)	Several ICERs	Semaglutide was cost-effective vs canagliflozin
Steen Carlsson & Persson 2014^251^ Sweden	T2DM	Liraglutide vs sulphonylurea or sitagliptin	Markov model	5	40 years	Societal	3%	Clinical trials	EQ-5D (Bagust & Beale)	Several ICERs	The cost per QALY forliraglutide was in the range considered medium by Swedish authorities
Stein *et al.* 2013^252^ US	DME	laser vs laser + ranibizumab vs laser + bevacizumab vs laser + triamcinolone	Markov model	18	25 years	Societal	3%	Clinical trial	TTO	Several ICERs	Bevacizumab therapy confers the greatest value among the different treatment options
Su *et al.* 2019^253^ China	T2DM	Insulin glargine vs insulin degludec	Simulation model	3	Lifetime	Health care payer	3%	Meta-analysis	EQ-5D (Beaudet *et al.* 2014)	Dominant	Insulin glargine is a cost-saving option compared with insulin degludec
Torre *et al.* 2020^254^ Italy	T2DM	Saxagliptin + dapagliflozinvs SOC	Simulation model	18	4 years	Health care payer	Not reported	Observational study (CVD-REAL)	Unknown	€11,517/QALY and €4639/QALY	Saxagliptin/dapagliflozin can be considered cost-effective
Tzanetakos *et al.* 2014^255^ Greece	T2DM	Liraglutide vs sitagliptin or exenatide	Simulation model	3	Lifetime	Health care payer	3.5%	Clinical trial (LEAD 6)	EQ-5D	Sitagliptin: €15,101/QALY Exenatide: €6818/QALY	Liraglutide is likely to be cost-effective
Tzanetakos *et al.* 2016^256^ Greece	T2DM	Dapagliflozin vs sulfonylurea or DPP-4-inhibitors	Microsimulation	2	Lifetime	Health care payer	3.5%	Clinical trial (Nauck *et al.*) + NMA (Barnett *et al.*)	EQ-5D	Sulfonylurea: €10,623/QALY DPP-4: €17,695/QALY	Dapagliflozin in combination with metformin was shown to be a cost-effective treatment
Tzanetakos *et al.* 2018^257^ Greece	T2DM	Exenatide vs insulin glargine or liraglutide	Simulation model	2	40 years	Health care payer	3.5%	Clinical trial (DURATION-3) + NMA (Scott *et al.*)	Unknown	Insulin glargine: €4499 Liraglutide: €2827	Exenatide was estimated to be cost-effective
Valentine *et al.* 2011a^258^ Sweden	T1DM	Insulin determir vs NPH	Semi-Markov	3	50 years	Societal	3%	Clinical trial (Farnkvist)	EQ-5D	SEK 49,757/QALY	Insulin detemir is likely to be cost-effective
Valentine *et al.* 2011b^259^ Switzerland, Denmark, Norway, Finland, Netherlands, and Austria	T2DM	Liraglutide vs exenatide	Simulation model	3	40 years	Health care payer	Several rates	Clinical trial (LEAD-6)	EQ-5D	Several ICERs	Liraglutide cost-effective
Valentine *et al.* 2012^260^ Denmark, Sweden, Finland, Netherlands	T1DM	Insulin detemir vs NPH insulin	No specification	16	1 year	Health care payer	No discounting	Meta-analysis	EQ-5D	Several ICERs	Insulin detemir is likely to be cost-effective vs NPH insulin
Valentine *et al.* 2018^261^ Germany	T1DM	Rapid-acting analog insulin regimen vs regular human insulin	Discrete event simulation model	8	50 years	Health care payer	3%	Meta-analysis (Singh et al)	EQ-5D	€4427/QALY	Rapid-acting analog insulin is likely to be considered cost-effective
Van der Linden *et al.* 2021^262^ Netherlands	T2DM	Dapagliflozin vs DPP-4 inhibitors	Microsimulation	2	40 years	Societal	4% for costs, 1.5% for effects	Clinical trial	EQ-5D	Dominant	Dapagliflozin is a cost-saving alternative to DPP-4 inhibitors
van Haalen *et al.* 2014^263^ Netherlands	T2DM	Dapagliflozin + insulin vs insulin	Simulation model	2	40 years	Societal	4% for costs, 1.5% for effects	Clinical trial	EQ-5D	€5502/QALY	Dapagliflozin in combination with insulin was estimated to be cost-effective
Vega-Hernandez *et al.* 2017^264^ UK	T2DM	Liraglutide vs dapagliflozin	Simulation model	3	Lifetime	Health care payer	3.5%	NMA	EQ-5D	Dominant and £11,857 to 13,227/QALY	Both dosages of liraglutide may be cost-effective treatment alternatives
Viljoen *et al.* 2019^265^ UK	T2DM	Semaglutide vs dulaglutide	Simulation model	3	Lifetime	Health care payer	3.5%	Clinical trial (SUSTAIN 7)	EQ-5D	Dominant	Compared with treatment with dulaglutide, semaglutide cost-effective
Viljoen *et al.* 2022^266^ UK	T2DM	Semaglutide vs dulaglutide	Simulation model	3	Lifetime	Health care payer	3.5%	ITC	EQ-5D	£228/QALY	Semaglutide cost-effective vs dulaglutide
Viriato *et al.* 2014^267^ Portugal	T2DM	Metformin + vildagliptin vs metformin + sulphonylurea	Simulation model	13	Lifetime	Health care payer	5%	Clinical trial	EQ-5D	€9072/QALY	Treatment with metformin + vildagliptin compared with metformin + sulphonylureis cost-effective
Wan *et al.* 2019^268^ China	T2DM	Bariatric surgery vs medication therapy	Markov model	18	40 years	Health care payer	5%	Observational study	EQ-5D	Dominant	Bariatric surgery is dominant
Watada *et al.* 2020^269^ Japan	T2DM	Linagliptin + SOC vs SOC	Microsimulation	18	Lifetime	Health care payer	2%	Clinical trial (CARMELINA)	EQ-5D	Dominant	The cost-effectiveness of linagliptin for a lifetime outcome was favorable
Yang *et al.* 2012^270^ China	T2DM	Insulin glargine vs insulin detemir	Simulation model	3	30 years	Health care payer	3%	Observational study	EQ-5D	Dominant	Conversion to insulin detemir from insulin glargine was cost-saving
Zhang *et al.* 2016^271^ China	T2DM	Liraglutide + metformin vs exenatide + metformin	Simulation model	3	30 years	Societal	3%	Observational study	Unknown	–11,550 RMB/QALYs	Liraglutide was superior to exenatide
Zupa *et al.* 2021^272^ US	T2DM	Semaglutide vs empagliflozin	Markov model	18	3 years	Health care payer	3%	Clinical trials (EMPA REG- and SUSTAIN)	EQ-5D (Zhang)	$19,964/QALY	Semaglutide is likely more cost-effective than empagliflozin

BIAsp 30, biphasic insulin aspart 30; CADTH Canadian Agency for Drugs and Technologies in Health; CVD, cardiovascular disease; DME, diabetic macular edema; DPP-4, dipeptidyl peptidase 4; DR, diabetic retinopathy; GLP-1 RA, glucagon-like peptide-1 receptor agonist; HUI3, Health Utilities Index Mark 3; ICER, incremental cost-effectiveness ratio; ITC, indirect treatment comparison; N/A, not applicable; NMA, network meta-analysis; NPH, neutral protamine Hagedorn; PROM, patient-reported outcome measure; QALY, quality-adjusted life year; QW, once weekly; RCT, randomized controlled trial; SGLT2, sodium-glucose cotransporter-2; SLR, systematic literature review; SOC, standard of care; T1DM, type 1 diabetes mellitus; T2DM, type 2 diabetes mellitus; TTO, time trade-off; VFQ-25, Visual Function Questionnaire 25; VFQ-UI, Visual Function Questionnaire, Utility index; TZD, thiazolidinediones.

^a^Models: 1) McEwan *et al.* 2016, 2) McEwan *et al.* 2015, 3) McEwan *et al.* 2014, 4) Hayes *et al.* 2013, 5) Lundqvist *et al.* 2014, 6) Wu *et al.* 2018, 7) Willis *et al.* 2017, 8) Valentine *et al.* 2017, 9) Brändle *et al.* 2011, 10) A model conducted by National Institute of Health and Care Excellence, 11) Eddy & Schlessinger 2003, 12) Kansal *et al.* 2019, 13) Viriato *et al.* 2014, 14) Ericsson *et al.* 2013, 15) Ridderstråle *et al.* 2013, 16) Valentine *et al.* 2012, 17) Abushanab *et al.* 2022a, 18) self-made model by authors, 19) health technology appraisal conducted by the National Institute for Health and Care Excellence or CADTH.

^b^Currency: CHF, Swiss francs; CNY, Chinese yuan; CZK, Czech koruna; DKK, Danish krone; HKD, Hong Kong dollar; JPY, Japanese yen; PLN, Polish złoty; QAR, Qatari riyal; RMB, renminbi (China); RSD, Servian dinar; SEK, Swedish krona; THB, Thai baht.

## Appendix V: Characteristics of the cost-effectiveness models, per condition (type 1 or 2 diabetes mellitus, diabetic retinopathy, or diabetic macular edema)

**Table:**

Characteristics	Type 1 diabetes mellitus (n=24)	Type 2 diabetes mellitus (n=208)	Diabetic retinopathy (n=1)	Diabetic macular edema (n=22)
*Year of publication*				
2011	1 (4%)	7 (3%)	0 (0%)	0 (0%)
2012	2 (8%)	15 (7%)	0 (0%)	2 (9%)
2013	1 (4%)	7 (3%)	0 (0%)	3 (14%)
2014	0 (0%)	9 (4%)	0 (0%)	2 (9%)
2015	2 (8%)	13 (6%)	0 (0%)	3 (14%)
2016	2 (8%)	18 (9%)	0 (0%)	3 (14%)
2017	5 (21%)	19 (9%)	0 (0%)	0 (0%)
2018	4 (17%)	18 (9%)	0 (0%)	1 (5%)
2019	1 (4%)	26 (13%)	1 (100%)	1 (5%)
2020	5 (21%)	22 (11%)	0 (0%)	2 (9%)
2021	0 (0%)	24 (12%)	0 (0%)	2 (9%)
2022	1 (4%)	30 (14%)	0 (0%)	3 (14%)
*Country*				
United Kingdom	8 (33%)	40 (19%)	0 (0%)	9 (41%)
United States	0 (0%)	30 (14%)	1 (100%)	6 (27%)
Canada	0 (0%)	8 (4%)	0 (0%)	1 (5%)
Japan	0 (0%)	6 (3%)	0 (0%)	0 (0%)
China	0 (0%)	32 (15%)	0 (0%)	2 (9%)
Nordics^a^	6 (25%)	22 (11%)	0 (0%)	1 (5%)
Latin America^b^	0 (0%)	7 (3%)	0 (0%)	0 (0%)
Europe^c^	8 (33%)	48 (23%)	0 (0%)	2 (9%)
Others^d^	2 (8%)	19 (9%)	0 (0%)	0 (0%)
Unknown	0 (0%)	1 (0.5%)	0 (0%)	1 (5%)
*Study perspective*				
Health care payer	20 (83%)	186 (89%)	1 (100%)	16 (73%)
Private health care payer	0 (0%)	1 (0.5%)	0 (0%)	0 (0%)
Provider	1 (4%)	1 (0.5%)	0 (0%)	0 (0%)
Societal	3 (13%)	24 (12%)	0 (0%)	6 (27%)
Unknown	0 (0%)	1 (0.5%)	0 (0%)	1 (5%)
*Time horizon*				
≤1 year	12 (50%)	17 (8%)	0 (0%)	0 (0%)
>1 and <10 years	1 (4%)	12 (6%)	0 (0%)	3 (14%)
≥10 and <30 years	1 (4%)	10 (5%)	1 (100%)	11 (50%)
≥30 years	9 (38%)	167 (80%)	0 (0%)	7 (32%)
Unknown	1 (4%)	4 (2%)	0 (0%)	1 (5%)
*Model type*				
Markov model (state-transition model)	3 (13%)	25 (12%)	0 (0%)	19 (86%)
Semi-Markov model	2 (8%)	8 (4%)	0 (0%)	0 (0%)
Simulation model	6 (25%)	134 (64%)	1 (100%)	0 (0%)
Discrete event simulation model	2 (8%)	9 (4%)	0 (0%)	0 (0%)
Microsimulation model	0 (0%)	14 (7%)	0 (0%)	1 (5%)
Partitioned-survival model	1 (4%)	0 (0%)	0 (0%)	0 (0%)
Decision tree	1 (4%)	3 (1%)	0 (0%)	0 (0%)
No specification	9 (38%)	15 (7%)	0 (0%)	2 (9%)
*Model*				
Cardiff T1DM^273^	1 (4%)	0 (0%)	0 (0%)	0 (0%)
Cardiff T2DM^274^	0 (0%)	28 (13%)	0 (0%)	0 (0%)
IQVIA CORE Diabetes Model^275^	6 (25%	87 (42%)	0 (0%)	0 (0%)
United Kingdom Prospective Diabetes Study Outcomes Model 2 (UKPDS-OM2)^276^	0 (0%)	14 (7%)	0 (0%)	0 (0%)
Swedish Institute for Health Economics Cohort Model for T2DM (IHECM T2DM)^277^	0 (0%)	12 (6%)	0 (0%)	0 (0%)
Chinese Outcomes Model for T2DM (COMT)^278^	0 (0%)	6 (3%)	0 (0%)	0 (0%)
Economic and Health Outcomes Model of T2DM (ECHO-T2DM)^279^	0 (0%)	2 (1%)	0 (0%)	0 (0%)
The PRIME model^280^	1 (4%)	0 (0%)	0 (0%)	0 (0%)
Cardiff Research Consortium Discrete Event Simulation (CRC DES) model^43^	1 (4%)	1 (0.5%)	0 (0%)	0 (0%)
Model by NICE	0 (0%)	0 (0%)	0 (0%)	2 (9%)
The Archimedes model^281^	0 (0%)	1 (0.5%)	0 (0%)	0 (0%)
Model by Kansal^139^	0 (0%)	4 (2%)	0 (0%)	0 (0%)
Model by Viriato^267^	0 (0%)	2 (1%)	0 (0%)	0 (0%)
Model by Ericsson^77^	7 (29%)	9 (4%)	0 (0%)	0 (0%)
Model by Ridderstråle^231^	2 (8%)	3 (1%)	0 (0%)	0 (0%)
Model by Valentine^260^	3 (13%)	2 (1%)	0 (0%)	0 (0%)
Model by Abushanab^31^	0 (0%)	2 (1%)	0 (0%)	0 (0%)
Model self-made by authors	4 (17%)	33 (16%)	1 (100%)	15 (68%)
NICE/CADTH health technology appraisal	1 (4%)	5 (2%)	0 (0%)	5 (23%)
*General PROM*				
EQ-5D	13 (54%)	159 (76%)	0 (0%)	5 (23%)
Time trade-off	4 (17%)	6 (3%)	1 (100%)	15 (68%)
Health Utilities Index	0 (0%)	4 (2%)	0 (0%)	1 (5%)
Standard gamble	0 (0%)	1 (0.5%)	0 (0%)	0 (0%)
Visual Function Questionnaire-25	0 (0%)	0 (0%)	0 (0%)	2 (9%)
Visual Function Questionnaire-Utility Index	0 (0%)	0 (0%)	0 (0%)	1 (5%)
Unclear	8 (33%)	41 (20%)	0 (0%)	2 (9%)

NOTE: Values may not total 100% because some publications included several choices.

CADTH Canadian Agency for Drugs and Technologies in Health; NICE, National Institute for Health and Care Excellence; PROM, patient-reported outcome measures; T1D, type 1 diabetes mellitus; T2DM, type 2 diabetes mellitus.

^a^Nordics: Denmark, Finland, Norway, Sweden.

^b^Latin America: Argentina, Brazil, Colombia, Ecuador, Mexico.

^c^Europe: Austria, Bulgaria, Czech Republic, Estonia, France, Germany, Greece, Italy, Netherlands, Poland, Portugal, Serbia, Slovakia, Spain, Switzerland.

^d^Other countries: Algeria, Australia, Hong Kong, India, Indonesia, Iran, Malaysia, Saudi Arabia, Singapore, South Korea, Taiwan, Thailand, Vietnam, Qatar.
