# Supplementary material for: Adverse drug events in cost-effectiveness models of pharmacological interventions for diabetes, diabetic retinopathy, and diabetic macular edema: a scoping review
Source: JBI Evid Synth. 2024 Jul 29;22(11):2194–266. doi: 10.11124/JBIES-23-00511 (PMC11554252; doi:10.11124/JBIES-23-00511)
Supplement: Supplementary file 2 [file srx-22-2194-s002.pdf]

JBI EVIDENCE SYNTHESIS

SUPPLEMENTAL FILE

The Finnish-language translation of this review abstract is presented as supplied by the author/translator. Readers should note that the translation has not been verified by *JBI Evidence Synthesis* or peer reviewed. In the event of any discrepancies, readers should refer to the English-version of this manuscript.

Vastuuvapauslauseke: Tämän katsauksen tiivistelmän suomenkielinen käännös on tekijän/kääntäjän toimittama. Lukijoiden on huomattava, että JBI Evidence Synthesis ei ole vahvistanut käännöstä eikä sitä ole vertaisarvioitu. Jos ristiriitaisuuksia ilmenee, lukijoiden on viitattava tämän tiivistelmän ja käsikirjoituksen englanninkieliseen versioon.

Pesonen M, Jylhä V, Kankaanpää E. Adverse drug events in cost-effectiveness models of pharmacological interventions for diabetes, diabetic retinopathy, and diabetic macular edema: a scoping review. *JBI Evidence Synthesis*. 2024. https://doi.org/10.11124/JBIES-23-00511

Translated by: Mari Pesonen & Eila Kankaanpää, August 1, 2024.

JBI EVIDENCE SYNTHESIS

Lääkehoitojen haittavaikutukset diabeteksen, diabeettisen retinopatian ja diabeettisen makulaturvotuksen kustannus-vaikuttavuusmalleissa: kartoittava katsaus

Tiivistelmä

Tavoite: Tämän katsauksen tavoitteena oli tarkastella, miten lääkehoitojen haittavaikutukset huomioidaan diabeteksen, diabeettisen retinopatian ja diabeettisen makulaturvotuksen hoitoon käytettävien lääkehoitojen kustannus-vaikuttavuusmalleissa.

Tutkimuksen tausta: Taloudellisen arvioinnin menetelmäsuosituksissa lääkehoitojen haittavaikutusten huomioimisen tärkeys tuodaan esille, mutta käytännössä lääkehoitojen haittavaikutusten huomioiminen kustannus-vaikuttavuusanalyyseissä on puutteellista. Lääkehoitojen haitallisten vaikutusten puutteellinen huomiointi voi vaikuttaa analyysin tulosten luotettavuuteen ja siksi taloudellisen arvioinnin tuottama tieto voi olla harhaanjohtavaa. Jotta ymmärretään paremmin, miten taloudellinen arviointi toteutetaan käytännössä, on tärkeää tutkia huomioidaanko lääkehoitojen haittavaikutukset ja miten ne huomioidaan kustannus-vaikuttavuusanalyyseissä.

Sisäänottokriteerit: Katsaukseen otettiin mukaan mallintamalla toteutettuja diabeteksen, diabeettisen retinopatian tai diabeettisen makulaturvotuksen lääkehoidon kustannus-vaikuttavuusanalyysejä , jotka oli julkaistu vuosina 2011–2022 englannin kielellä. Muut taloudellisen arvioinnin analyysimenetelmät ja sairaudet poissuljettiin katsauksesta.

Menetelmät: Katsauksen julkaisut haettiin MEDLINE (PubMed), CINAHL (EBSCOhost), Scopus, Web of Science Core Collection, ja NHS Economic Evaluation Database -tietokannoista. Harmaata kirjallisuutta haettiin the National Institute for Health and Care Excellence, European Network for Health Technology Assessment, the National Institute for Health and Care Research, ja International Network of Agencies for Health Technology Assessment -tietokannoista. Tiedonhaku toteutettiin 1.1.2023. Kaksi arvioijaa toteutti otsikoiden ja abstraktien seulonnan, ja kolme arvioijaa toteutti kokotekstien arvioinnin. Tietojen poiminta ja analysointi toteutettiin tähän tarkoitukseen suunnitellulla lomakkeella. Tulokset esitettiin taulukkoina ja sanallisena yhteenvetona. Johtopäätöksissä katsauksen tuloksia tarkasteltiin suhteessa olemassa olevaan kirjallisuuteen ja taloudellisen arvioinnin menetelmäsuosituksiin.

Tulokset: Tässä katsauksessa mukana oli yhteensä 242 julkaistua analyysiä. Tyypin 2 diabetes oli yleisin sairaus mukaan otetuissa analyyseissä (86 %), tyypin 1 diabeteksen (10 %), diabeettisen

makulaturvotuksen (9 %), ja diabeettisen retinopatian (0.4 %) osuudet olivat pienempiä. Suurin osa mukaan otetuista analyyseistä oli toteutettu terveydenhuollon näkökulmasta (88 %) ja niiden aikahorisontti oli 30 vuotta tai pidempi (75 %). Yleisin mallinnusmenetelmä oli simulaatiomalli (57 %), ja toiseksi yleisin Markov-simulaatiomalli (18 %). Mukaan otetuista analyyseistä 25 % huomioi lääkehoitojen haittavaikutukset, kun taas 13 % ei huomioinut niitä. Suurin osa (61 %) mukaan otetuista analyyseistä huomioi lääkehoitojen haittavaikutukset osittain, mikä tarkoittaa sitä, että vain yksi tai kaksi lääkehoitojen haittavaikutusta oli huomiotu analyysissä. Haittavaikutusten huomioimisessa ei ollut merkittäviä eroja sairausryhmien välillä, mutta diabeettisen retinopatian ja diabeettisen makulaturvotuksen lääkehoidoille toteutetut mallit jättivät lääkehoitojen haittavaikutuksiin liittyvän elämänlaatuvaikutuksen useammin huomioimatta verrattuna diabeteksen lääkehoidoille toteutettuihin malleihin. Suurin osa mukaan otetuista analyyseistä huomioi lääkehoitojen haittavaikutukset malleissa todennäköisyyksinä (55 %) tai alamallina (42 %). Yleisimmin tieto lääkehoitojen haittavaikutusten ilmaantuneisuudesta perustui kliinisiin tutkimuksiin (65 %).

Johtopäätökset: Lääkehoitojen huomiointi kustannus-vaikuttavuusmalleissa ei ole ihanteellista. Tämän katsauksen tulosten mukaan lääkehoitojen haittavaikutuksiin liittyvät kustannukset oli huomioitu analyyseissä paremmin verrattuna lääkehoitojen haittavaikutusten elämänlaatuun. Erityisesti tämä korostui diabeettisen retinopatian ja diabeettisen makulaturvotuksen lääkehoidoille toteutetuissa malleissa. Lääkehoitojen haittavaikutusten mahdollista vaikutusta kustannus-vaikuttavuusanalyysin tuloksiin olisi hyvä tulevaisuudessa tutkia lisää. On tärkeää tunnistaa kriteerit ja keinot, joiden avulla lääkehoitojen haittavaikutukset voitaisiin huomioida taloudellisen arvioinnin käytännön toteutuksessa.

Asiasanat: lääkehoitojen haittavaikutukset; kustannus-vaikuttavuusanalyysi; diabetes; diabeettinen makulaturvotus; diabeettinen retinopatia
